# Synthesis and Evaluation of New β-Carboline-3-(4-benzylidene)-*4H*-oxazol-5-one Derivatives as Antitumor Agents

**DOI:** 10.3390/molecules17056100

**Published:** 2012-05-21

**Authors:** Franciele Cristina Savariz, Mary Ann Foglio, João Ernesto de Carvalho, Ana Lúcia T. G. Ruiz, Marta C. T. Duarte, Mauricio Ferreira da Rosa, Emerson Meyer, Maria Helena Sarragiotto

**Affiliations:** 1 Departamento de Química, Centro de Ciências Exatas, Universidade Estadual de Maringá, Av. Colombo, 5790, Maringá, 87020-900 PR, Brazil; 2 Centro Pluridisciplinar de Pesquisas Químicas, Biológicas e Agrícolas (CPQBA), Universidade Estadual de Campinas, 6171, Campinas, 13083-970 SP, Brazil; 3 Centro de Engenharias e Ciências Exatas, Universidade Estadual do Oeste do Paraná, Rua da Faculdade, 645, Toledo, 85903-000 PR, Brazil

**Keywords:** β-carboline, *4H*-oxazol-5-one, cytotoxic activity, antimicrobial activity

## Abstract

In the present work, we report the synthesis and *in vitro* anticancer and antimicrobial activity evaluation of a new series of 1-substituted-β-carboline derivatives bearing a 4-benzylidene-*4H-*oxazol-5-one unity at C-3. The compound 2-[1-(4-methoxyphenyl)-9H-β-carbolin-3-yl]-4-(benzylidene)*-4H-*oxazol-5-one (**11**) was the most active derivative, exhibiting a potent cytotoxic activity against glioma (U251), prostate (PC-3) and ovarian (OVCAR-03) cancer cell lines with IC_50_ values of 0.48, 1.50 and 1.07 µM, respectively. An *in silico* study of the ADME properties of the novel synthesized β-carboline derivatives was also performed.

## 1. Introduction

Natural and synthetic tetrahydro-β-carbolines and β-carbolines are a class of alkaloids with a large spectrum of important pharmacological properties [1,2,3,4,5,6,7]. Among the activities presented, the antitumor activity has received special attention, and several studies on structure-activity relationship of β-carbolines have focused their anticancer activities [8−16]. The SAR studies have demonstrated that the introduction of appropriate substituents into the positions −1, −2, −3 and −9 of the β-carboline nucleus resulted in more potent antitumor β-carboline derivatives, with reduced toxicity. 

The potential of β-carbolines and the importance of the search for new antitumor agents led our group to a continuing study of this class of compounds. In previous works, we reported the synthesis and *in vitro* antitumor activities, against a panel of human cancer cell lines, of a series of 1-substituted β-carboline derivatives bearing different substituents at C-3, such as 2-substituted-1,3,4-oxadiazole and 5-substituted-1,2,4-triazole rings [17], 3-alkylamino(methyl)-2-thioxo-1,3,4-oxadiazole groups [18] and *N*-(substituted-benzylidene)-carbohydrazide groups [19]. The anticancer assay results indicated several compounds with potent anticancer activity, with IC_50_ values lower than 1.0 μM for some of the human cancer cell lines tested [17,18,19]. In addition to the antitumor activity, β-carbolines containing the 2-thioxo-1,3,4-oxadiazole group at C-3 exhibited antimicrobial activity towards the fungus *Candida albicans* and the bacterium *Bacillus subtilis* [18]. Antimicrobial activity of β-carboline derivatives, mainly against *Bacillus subtilis*, *Escherichia coli* and *Staphylococcus aureus* bacteria and against *Candida albicans* fungi, was also reported in literature [20,21].

With the aim of evaluating the influence of different substituents at position-3 of 1-substituted-β-carbolines, in this work we propose the incorporation of a benzylidene-*4H-*oxazol-5-one unity at C-3, expecting an improvement of the antitumor and antimicrobial activities in relation to the most active β-carboline derivatives reported in our previous work [17,18,19]. The *4H*-oxazol-5-one ring is found in natural and synthetic compounds possessing important biological activities, such as antimicrobial [22,23,24], antiviral [25], antiangiogenic [26], inhibitory of tyrosinase [27], cytotoxic and immunomodulatory properties [28]. Due their biological importance, several methods were reported for the synthesis of oxazolones [29,30,31].

Thus, in the present work, we report the synthesis and *in vitro* cytotoxic and antimicrobial activity evaluation of some novel 1-substituted β-carboline derivatives bearing a 4-(benzylidene)-*4H-*oxazol-5-one moiety at C-3. Additionally, an *in silico* study of the ADME properties of the novel synthesized β-carboline derivatives was carried out by investigating their Lipinski’s parameters, topological polar surface area (TPSA) and percentage of absorption (% ABS). 

## 2. Results and Discussion

### 2.1. Chemistry

**Scheme 1:**
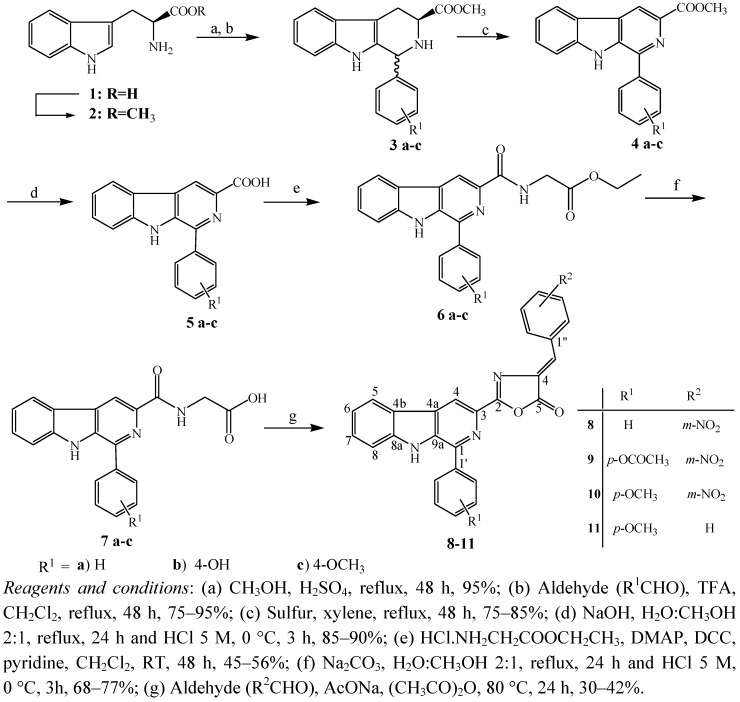
The synthetic route to compounds **8–11**.

The synthetic route for the β-carboline-3-(4-benzylidene)-*4H-*oxazol-5-ones **8–11** is outlined in Scheme 1. The 1-substituded β-carboline-3-carboxylic acids **5a–c** were prepared from commercial L-tryptophan (**1**), according to the synthetic protocol described by our group [17,18,19]. The Pictet-Spengler condensation of L-tryptophan methyl ester (**2**) with benzaldehyde, 4-hydroxybenzaldehyde and 4-methoxybenzaldehyde afforded the 1,2,3,4-tetrahydro-β-carbolines **3a–c**. Oxidation of **3a–c** with sulfur in refluxing xylene, led to the methyl β-carboline-3-carboxylates **4a–c**, which were hydrolyzed under basic conditions to give the β-carboline-3-carboxylic acids **5a–c**. 

In order to synthesize the β-carboline-3-(4-benzylidene)-*4H-*oxazol-5-ones **8–11**, the Erlenmeyer-Plöchl reaction, the most common route to oxazolones, was employed [31]. The derivatives **5a–c** were converted to the respective *N*-(1-benzylidene-β-carboline-3-carbonyl)-glycine ethyl esters **6a–c** by activation of the β-carboline-3-carboxylic acids **5a–c** with *N*,*N'*-dicyclohexylcarbodiimide (DCC) and dimethylaminopyridine (DMAP), in pyridine, followed by treatment with glycine ethyl ester hydrochloride [32]. To prepare the β-carboline-3-carbonyl-amino acids **7a–c**, the corresponding esters **6a–c** were submitted to hydrolysis with sodium carbonate in refluxing methanol/water. Erlenmeyer-Plöchl reaction of **7a** and **7c**with 3-nitrobenzaldehyde or benzaldehyde, afforded the corresponding β-carboline-3-oxazolones **8–11**. The reaction of the derivative **7b**, bearing the 4-hydroxyphenyl substituent at C-1, led to the oxazolone **9** with the hydroxyl group acetylated.

To evaluate the effects of electron-donating groups at the R^2^ position of the benzylidene-*4H-*oxazol-5-one moiety on activity, compounds **7a** and **7c** were subjected to Erlenmeyer-Plöchl reaction with 4-*N*,*N*-dimethylaminobenzaldehyde and 4-methoxybenzaldehyde. However, these reactions failed to furnish the corresponding β-carboline-3-oxazolones, probably due to the relative low electrophilicity of the aldehydes employed.

All novel compounds were characterized by their spectroscopic data (IR, HR-ESIMS, ^1^H and ^13^C-NMR), which are described in the Experimental section. The (4-benzylidene)-*4H-*oxazol-5-one group at C-3 of **8–11** was evidenced by the presence of a singlet at δ_H_ 7.54–7.56 in the ^1^H-NMR spectra, corresponding to the benzylidene hydrogen, which showed a correlation with the carbon signals at δ_C_ 126.4–131.5, in the HSQC spectra. The signals at δ_C_ 164.0–164.5 and 168.0–166.9, in the ^13^C-NMR spectra, were assigned to the C-2 and C-5 carbons, respectively, of the *4H*-oxazol-5-one ring. The IR spectra showed absorption bands characteristic for C=O stretching in the 1,780–1,816 cm^−1^ region.

The structures of **8–11** were also confirmed by their HR-ESI and EI mass spectra. The compounds showed the presence of the molecular ions [M^+^] consistent with the expected structures, and a base peak at *m/z* [M^+^−substituted-benzylidene oxazolone] corresponding to the cleavage between C-3 of β-carboline and C-2 of the *4H*-oxazol-5-one ring.

### 2.2. Cytotoxic Activity

**Figure 1:**
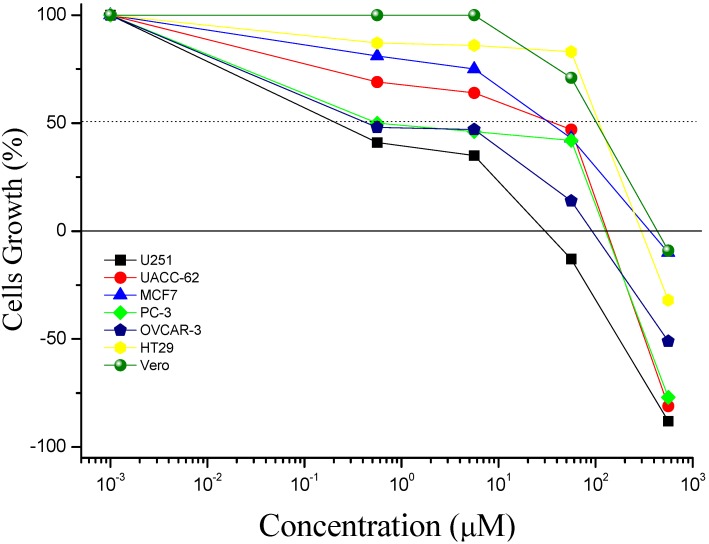
Concentration (μM) *versus* cell growth (%) for compound **11**.

The IC_50_ values obtained for the synthesized compounds are shown in Table 1. Analysis of the IC_50_ values (Figure 1, Table 1) showed that compound **8** was active only against the melanoma (UACC-62) cell line, with IC_50_ of 7.52 μM. The same result was observed for the derivative **9**, where the phenyl group at C-1 was changed for a 4-acetoxyphenyl group.

On the other hand, the substitution of the phenyl group at C-1, in compound **8**, for a 4-methoxy-phenyl group resulted in the more active compound **10**, showing that a good electron-donating substituent at the phenyl ring is important for the cytotoxicity. A potent activity against glioma (U251) and ovarian (OVCAR-03) cell lines was observed for compound **10**, which presented IC_50_ values of 0.35 and 2.18 μM, respectively. Keeping the 4-methoxyphenyl group at C-1 and in order to evaluate the effect on activity of other groups at the oxazolone moiety, the 3-nitrophenyl group was substituted for a phenyl group, resulting in compound **11**, which displayed the better activity in comparison to all other synthesized compounds. Derivative **11** showed IC_50_ values ≤ 10.00 μM for four of the cells lines tested (Table 1). A potent activity was observed mainly against glioma (U251), prostate (PC-3) and ovarian (OVCAR-03) cell lines with IC_50_ values of 0.48, 1.50 and 1.07 μM, respectively.

**Table 1:** IC_50_ values (in μM) for compounds **8–11**.

	Glioma U251	Melanoma UACC-62	Breast MCF7	Prostate PC-3	Ovarian OVCAR-03	Colon HT-29	VERO
**Doxorubicin** **^a^**	0.03	0.04	0.06	0.05	0.42	0.18	0.50
**8**	62.42	7.52	>100	>100	63.19	>100	>100
**9**	80.20	8.76	>100	>100	>100	>100	>100
**10 **	0.35	15.93	47.63	5.28	2.18	>100	>100
**11**	0.48	10.00	23.44	1.50	1.07	67.88	63.17

^a^ Doxorubicin was the positive control.

In addition to the effective growth inhibition (IC_50_ values), compound **11** showed also cytostatic activity, with IC_100_ values of 15.50, 56.83, 64.17 and 112.42 μM for glioma (U251), ovarian (OVCAR-03), prostate (PC-3) and melanoma (UACC-62) human cell lines, respectively (Figure 1). 

The comparison of IC_50_ data of **11** (Table 1) with those of the most active β-carboline derivatives reported in our previous work [17,18,19] demonstrated, in general, a lower cytotoxic activity for this compound. However, specifically for the prostate (PC-3) and ovarian (OVCAR-03) cancer cell lines, the cytotoxic activity of **11** was similar to that of some active reported compounds, such as 1-(4-*N*,*N*-dimethylaminophenyl)-3-(5-thioxo-1,2,4-triazol-3-yl)-β-carboline (prostate: IC_50_ = 1.37 μM; ovarian: IC_50_ = 1.09 μM) [17] and *N'*-(2-chlorobenzylidene)-1-(4-hydroxyphenyl)-β-carboline-3-carbohydrazide (prostate: IC_50_ = 1.83 μM, ovarian: 1.65 μM) [19].

The IC_50_ data shown in Table 1 were also compared with those of previously reported β-carboline derivatives bearing the 4-methoxyphenyl group at position-1 and the 2-thioxo-1,3,4-oxadiazole, 2-methylthio-1,3,4-oxadiazole and 5-thioxo-1,2,4-triazole groups [17], and (substituted benzylidene)-carbohydrazide groups [19], at position-3. This comparison pointed of that, except for 1-(4-methoxyphenyl)-3-(2-methylthio-1,3,4-oxadiazol-5-yl) β-carboline (ovarian: IC_50_ = 2.13 μM) [17], the synthesized oxazoles **10** and **11** showed higher cytotoxic activity, particularly for prostate (PC-3) and ovarian (OVCAR-03) cancer cell lines, than all other 1-(4-methoxyphenyl)-3-substituted-β-carboline derivatives previously tested [17,19]. These data demonstrate the importance of the (substituted benzylidene)-*4H-*oxazol-5-one moiety on the cytotoxic activity of compounds **10** and **11**.

### 2.3. Antimicrobial Activity

Compounds **8–11** were assayed against the bacteria *Bacillus subtilis* ATCC 2576, *Escherichia coli* ATCC 25922, *Pseudomonas aeruginosa* ATCC 13388, *Staphylococus aureus* ATCC 6538, and against the fungi *Candida albicans* ATCC 10231, *Candida parapsilosis* ATCC-22019 and *Candida tropicalis* ATCC-28707. Contrarily to the expectation, the assay results showed no activity for compounds **8–11** (IC_50_ > 100 µM), indicating that the (substituted-benzylidene)-*4H-*oxazol-5-one group at C-3 did not contribute to the antimicrobial activity. 

### 2.4. *In Silico* Study

An *in silico* computational study of the synthesized β-carboline-3-(4-benzylidene)-*4H-*oxazol-5-ones **8–11** was performed by determining the Lipinski’s parameters, topological polar surface area (TPSA) and percentage of absorption (% ABS) [33,34,35]. Calculations were performed using the *Molinspiration Online Property Calculation Toolkit* (www.molinspiration.com) [34] and *OSIRIS Property Explorer* (www.organic-chemistry.org/prog/peo) [35]. The percentage of absorption was estimated using the equation: % ABS = 109 − 0.345 × TPSA [33]. These data are shown in Table 2. 

*In vivo* absorption of the new synthesized derivatives was tentatively assessed by means of theoretical calculations following Lipinski’s rule of five, which establishes that the absorption or permeation of an orally administered compound is more likely to be good if the drug satisfies the following criteria: (a) hydrogen bond donors ≤ 5 (OH and NH groups); (b) hydrogen bond acceptors ≤10 (N and O atoms); (c) molecular weight < 500; (d) calculated logP < 5 [33,34,35]. Compounds violating more than one of these rules may present bioavailability problems.

**Table 2:** Lipinsk’s parameters and % ABS, TPSA, LogS for compounds **8–11**.

Comp.			Lipinsk’s parameters					
	%ABS	TPSA ^a^ (A^2^)	nHBA (NO)	nHBD (OHNH)	logP ^a^	MW	n violations	logS ^a^
**8**	68.42	117.61	8	1	5.45	460.45	1	−7.41
**9**	59.35	143.91	10	1	5.00	518.48	1	−7.71
**10**	65.24	126.85	9	1	5.50	490.47	1	−7.43
**11**	81.05	81.02	6	1	5.57	445.48	1	−6.97

^a^ www.molinspiration.com; ^b^ www.organic-chemistry.org/prog/peo; %ABS = 109 − 0.345 × TPSA; Number hydrogen bond acceptor(NO)*= n*HBA ≤ 10; Number hydrogen bond donors (OHNH) =*n*HBD ≤ 5; MW ≤ 500; Octanol-water partition coefficient = LogP < 5; Solubility = LogS > −4.

Our results (Table 2) revealed that the β-carboline-3-(4-benzylidene)-*4H-*oxazol-5-ones **8–11** violated only one of the Lipinski’s rules. Compound **9** has a molecular weight larger than 500 g/moL and the derivatives **8**, **10** and **11** showed octanol-water partition coefficients (LogP) larger than 5.0.

All derivatives have number of hydrogen bond acceptors (*n*-ON = 6–10) and donors (*n*-OHNH = 1) in agreement with Lipinski’s rule. The calculated percent absorption (% ABS) of all derivatives ranged between 59.35 and 81.05%, indicating that these compounds should have good cellular plasmatic membrane permeability. 

## 3. Experimental

### 3.1. Chemistry

Melting points were determined in a Micro-Química MQAPF-301 apparatus and are uncorrected. ^1^H- and ^13^C-NMR spectra were recorded in a Varian model Mercury plus 300 spectrometer (Palo Alto, CA, USA) at 300 MHz and 75.5 MHz, respectively, with DMSO-*d_6_* as solvent and TMS as the internal standard. EI-MS spectra were recorded in a Thermoelectron Corporation Focus-DSQ II spectrometer (Austin, TX, USA). HR-ESI mass spectra were recorded on a Q-TOF (Micromass) spectrometer in positive ion mode (Manchester, UK). IR spectra were recorded on a BOMEM model MB‑100 spectrometer (Quebec, CA, USA). For TLC, Merck precoated plates (silica gel 60 G254) were used. Silica gel 60 Merck (230–400 mesh) was used in the column chromatography purification of some compounds. All reagents were purchased from commercial suppliers.

### 3.2. General Method for Synthesis of N-(1-benzylidene-β-carboline-3-carbonyl)-glycine Ethyl Esters **6a–c**

To a solution of β-carboline-3-carboxylic acids **5a–c** (0.7 mmol) in pyridine (5 mL) at 0 °C was added glycine ethyl ester hydrochloride (0.7 mmol), DMAP (0.07 mmol) in CH_2_Cl_2_ (5 mL). The mixture was stirred for 5 min and a solution of DCC (0.7 mmol) in CH_2_Cl_2_ (5 mL) was added, followed by stirring for 2 h, at 0 °C, and for 24 h at room temperature. This step was repeated with a new amount of DCC (0.7 mmol) in CH_2_Cl_2_ (5 mL), followed by evaporation of the solvent, addition of CH_2_Cl_2_ and filtration of the DCU precipitate formed. The solvent was removed from the filtrate and the residual DCU separated by precipitation with acetone and filtration. The solvent was removed and the residue obtained purified on a chromatographic column (silica gel, CHCl_3_/CH_3_OH 5%) to give the pure β-carbolines **6a–c** in yields ranging from 45–56%.

*N-(1-Phenyl-β-carboline-3-carbonyl)-glycine ethyl ester* (**6a**). Yield: 55%, m.p. 194–195 °C. IR (KBr) ν_max_ cm^−1^: 1737 (C=O); 1657 (C=O), 1622 (C=N); 1591–1494 (C=C). ^1^H-NMR (CDCl_3_): *δ* 8.80 (s, H-4), 8.18 (d, *J =* 7.8 Hz, H-5), 7.35 (td, *J =* 7.3 Hz and 1.5 Hz, H-6), 7.49–7.63 (m, H-7), 7.49–7.63 (m, H-8), 8.89 (s, NH-9), 7.99 (dd, *J =* 6.9 Hz and 1.5 Hz, H-2'), 7.51–7.64 (m, H-3'/5'), 7.49–7.64 (m, H-4'), 7.99 (dd, *J =* 6.9 Hz and 1.5 Hz, H-6'), 8.65 (t, *J =* 5.7 Hz, NH-2''), 4.31 (d, *J =* 5.7 Hz, NHCH_2_), 4.27 (q, *J =* 7.2 Hz, OCH_2_CH_3_), 1.33 (t, *J =* 7.2 Hz, CH_3_CH_2_O). ^ 13^C-NMR (CDCl_3_): *δ* 141.2 (C-1), 139.8 (C-3), 113.7 (C-4), 130.6 (C-4a), 122.4 (C-4b), 122.3 (C-5), 121.1 (C-6), 129.1 (C-7), 112.1 (C-8), 140.9 (C-8a), 137.9 (C-9a), 135.0 (C-1'), 128.4 (C-2'), 129.3 (C-3'/5'), 129.3 (C-4'), 128.4 (C-6'), 166.0 (C=O), 41.7 (NHCH_2_), 170.5 (C=O), 61.7 (OCH_2_CH_3_), 14.4 (CH_3_CH_2_O). EIMS, 70 eV, *m/z* (rel. int. %): 373 (40, M^+∙^), 243 (100), 271 (40).

*N-[1-(4-Hydroxyphenyl)-β-carboline-3-carbonyl]-glycine ethyl ester* (**6b**). Yield: 45%, m.p. 168–170 °C. IR (KBr) ν_max_ cm^−1^: 1729 (C=O), 1626 (C=N), 1538–1464 (C=C). ^1^H-NMR (CDCl_3_): *δ* 8.72 (s, H-4), 8.27 (d, *J =* 7.8 Hz, H-5), 7.28 (t, *J =* 7.8 Hz, H-6), 7.54 (t, *J =* 7.8 Hz, H-7), 7.68 (d, *J =* 7.8 Hz, H-8), 11.69 (s, NH-9), 8.01 (d, *J =* 8.7 Hz, H-2'/6'), 7.04 (d, *J =* 8.7 Hz, H-3'/5'), 9.71 (brs, OH), 8.97 (t, *J =* 6.0 Hz, NH-2''), 4.17 (d, *J =* 6.0Hz, NHCH_2_), 4.18 (q, *J =* 7.2 Hz, OCH_2_CH_3_), 1.27 (t, *J =* 7.2 Hz, CH_3_CH_2_O). ^13^C-NMR (CDCl_3_): *δ* 141.3 (C-1), 138.6 (C-3), 111.9 (C-4), 127.9 (C-4a), 121.1 (C-4b), 121.3 (C-5), 119.7 (C-6), 128.2 (C-7), 112.4 (C-8), 141.0 (C-8a), 133.9 (C-9a), 129.3 (C-1'), 129.8 (C-2'/6'), 115.3 (C-3'/5'), 158.2 (C-4'), 165.2 (C=O), 40.9 (NHCH_2_), 169.8 (C=O), 60.3 (OCH_2_CH_3_), 13.9 (CH_3_CH_2_O). EIMS, 70 eV, *m/z* (rel. int. %): 389 (25, M^+^), 259 (100), 343 (10).

*N-[1-(4-Methoxyphenyl-β-carboline-3-carbonyl]-glycine ethyl ester* (**6c**). Yield: 56%, m.p. 184–186 °C. IR (KBr) ν_max_ cm^−1^: 1742 (C=O); 1656 (C=O), 1609 (C=N), 1561–1464 (C=C). ^1^H-NMR (CDCl_3_): *δ* 8.79 (s, H-4), 8.19 (d, *J =* 7.8 Hz, H-5), 7.35 (td, *J =* 7.0 Hz; *J =* 1.5 Hz, H-6), 7.53–7.61 (m, H-7), 7.53–7.61 (m, H-8), 8.81 (s, NH-9), 7.95 (dd, *J =* 8.7 Hz and 1.8 Hz, H-2'/6'), 7.13 (dd, *J =* 8.7 Hz and 1.8 Hz, H-3'/5'), 3.92 (s, OCH_3_), 8.67 (t, *J =* 5.5 Hz, NH-2''), 4.32 (d, *J =* 5.5 Hz, NHCH_2_), 4.27 (q, *J =* 7.0 Hz, OCH_2_CH_3_), 1.33 (t, *J* *=* 7.0 Hz, CH_3_CH_2_O). ^13^C-NMR (CDCl_3_): *δ* 141.2 (C-1), 139.8 (C-3), 113.3 (C-4), 122.5 (C-4b), 122.3 (C-5), 121.1 (C-6), 128.9 (C-7), 112.0 (C-8), 140.8 (C-8a), 134.9 (C-9a), 130.5 (C-1'), 129.7 (C-2'/6'), 114.8 (C-3'/5'), 160.6 (C-4'), 55.6 (OCH_3_), 166.1 (C=O), 170.5 (C=O), 41.7 (NHCH_2_), 61.7 (OCH_2_CH_3_), 14.4 (CH_3_CH_2_O). EIMS, 70 eV, *m/z* (rel. int. %): 403 (60, M^+^), 301 (45), 273 (100), 258 (30).

### 3.3. General Method for the Synthesis of 1 N-(1-substituted-β-carboline-3-carbonyl)-glycine **7a–c**

A suspension of *N*-(1-substituted-β-carboline-3-carbonyl)-glycine ethyl esters **6a–c** (0.5 mmol) and Na_2_CO_3_ (1.5 mmol) in H_2_O:MeOH 2:1 (5 mL) was stirred for 24 h, at 80 °C. The mixture was cooled at 0 °C, and after stirring for 2 h, the solution was neutralized with a solution of 5 M HCl solution. The product was collected by filtration, dried and crystallized from ethanol, furnishing the compounds **7a–c** in 68–77% yield.

*N-(1-Phenyl-β-carboline-3-carbonyl)-glycine* (**7a**). Yield: 77%, m.p. 236–238 °C. IR (KBr) ν_max_ cm^−1^: 1731 (C=O); 1636 (C=N), 1593–1464 (C=C). ^1^H-NMR (DMSO-*d_6_*): *δ* 8.83 (s, H-4), 8.43 (d, *J =* 7.8 Hz, H-5), 7.32 (t, *J =* 7.8 Hz; H-6), 7.55–7.71 (m, H-7), 7.55–7.71 (m, H-8), 11.87 (s, NH-9), 8.14 (dd, *J* *=* 7.8 Hz and 1.2 Hz, H-2'/6'), 7.55–7.71 (m, H-3'/5'), 7.55–7.71 (m, H-4'), 8.87 (t, *J =* 5.1 Hz, NH-2''), 3.89 (d, *J =* 5.1 Hz, NHCH_2_). ^ 13^C-NMR (DMSO-*d_6_*): *δ* 141.6 (C-1), 139.5 (C-3), 112.9 (C-4), 129.9 (C-4a), 122.0 (C-4b), 121.2 (C-5), 120.2 (C-6), 128.9 (C-7), 112.7 (C-8), 140.6 (C-8a), 137.5 (C-9a), 134.2 (C-1'), 128.6 (C-2'/6'), 128.8 (C-3'/5'), 128.8 (C-4'), 164.7 (C=O), 41.9 (NHCH_2_), 171.4 (C=O). EIMS, 70 eV, *m/z* (rel. int. %): 345 (20, M^+^), 301 (20), 243 (95), 60 (100).

*N-[1-(4-Hydroxyphenyl)-β-carboline-3-carbonyl]-glycine* (**7b**). Yield: 74%, mp decomp. IR (KBr) ν_max_ cm^−1^: 1729 (C=O), 1609 (C=N), 1540–1498 (C=C). ^1^H-NMR (DMSO-*d_6_*): *δ* 8.76 (s, H-4), 8.40 (d, *J =* 7.6 Hz, H-5), 7.31 (t, *J =* 7.6 Hz, H-6), 7.59 (t, *J =* 7.6 Hz, H-7), 7.70 (d, *J =* 7.6 Hz, H-8), 12.64 (s, NH-9), 8.04 (d, *J =* 8.7 Hz, H-2'/6'), 7.04 (d, *J =* 8.7 Hz, H-3'/5'), 9.86 (brs, OH), 8.95 (t, *J* *=* 6.0 Hz, NH-2''), 4.08 (d, *J =* 6.0 Hz, NHCH_2_), 11.76 (s, OH). ^13^C-NMR (DMSO-*d_6_*): *δ* 141.5 (C_0_-1), 139.2 (C-3), 112.3 (C-4), 128.4 (C-4a), 121.3 (C-4b), 121.9 (C-5), 120.1 (C-6), 128.3 (C-7), 112.7 (C-8), 141.0 (C-8a), 133.9 (C-9a), 129.6 (C-1’), 130.1 (C-2'/6'), 115.6 (C-3'/5'), 158.4 (C-4'), 165.2 (C=O), 41.2 (NHCH_2_), 171.6 (C=O). C_20_H_15_N_3_O_4_ EIMS, 70 eV, *m/z* (rel. int. %): 361 (20, M^+^), 260 (100), 229 (20), 60 (10).

*N-[1-(4-Methoxyphenyl-β-carboline-3-carbonyl]-glycine* (**7c**). Yield: 68%, m.p. 247–249 °C. IR (KBr) ν_max_ cm^−1^: 1741 (C=O), 1638 (C=N), 1545–1464 (C=C). ^1^H-NMR (DMSO-*d_6_*): *δ* 8.76 (s, H-4), 8.28 (d, *J =* 7.5 Hz, H-5), 7.30 (t, *J =* 7.5 Hz, H-6), 7.56 (t, *J =* 7.5 Hz, H-7), 7.69 (d, *J =* 7.5 Hz, H-8), 11.74 (s, NH-9), 8.11 (d, *J =* 8.7 Hz, H-2'/6'), 7.91 (d, *J =* 8.7 Hz, H-3'/5'), 3.91 (s, OCH_3_), 8.90 (t, *J =* 5.7 Hz, NH-2''), 4.13 (d, *J =* 5.7 Hz, NHCH_2_). ^13^C-NMR (DMSO-*d_6_*): *δ* 141.5 (C-1), 138.6 (C-3), 112.3 (C-4), 121.1 (C-4b), 121.4 (C-5), 119.9 (C-6), 128.1 (C-7), 112.5 (C-8), 140.4 (C-8a), 134.0 (C-9a), 129.5 (C-1'), 129.9 (C-2'/6'), 113.8 (C-3'/5'), 159.8 (C-4'), 55.0 (OCH_3_), 164.8 (C=O), 40.9 (NHCH_2_), 171.2 (C=O). EIMS, 70 eV, *m/z* (rel. int. %): 375 (25, M^+∙^), 274 (100), 229 (25), 60 (40).

### 3.4. General Method for Synthesis of 1-Substituted β-carboline-3-(4-benzylidene)-4H-oxazol-5-ones **8–11**

To a solution of derivatives **7a–c** (0.3 mmol), in acetic anhydride (5 mL), was added 4-nitro-benzaldehyde or benzaldehyde (0.75 mmol), followed by addition of sodium acetate (1.5 mmol). The solution was stirred for 24 h at 80 °C and then, for 1h at 0 °C. The solid formed was filtered under vacuum and washed with cold acetone to afford the compounds **8–11**.

*2-(1-Phenyl-9H-β-carbolin-3-yl)-4-(3-nitrobenzylidene)-4H-oxazol-5-one* (**8**). Yield: 42%, m.p. 271–273 °C. IR (KBr) ν_max_ cm^−1^: 3395 (N–H), 1810 (C=O); 1625 (C=N), 1547–1497 (C=C). ^1^H-NMR (DMSO-*d_6_*): *δ* 9.17 (s, H-4), 8.49 (d, *J =* 7.8 Hz, H-5), 7.41 (t, *J =* 7.8 Hz, H-6), 7.59–7.66 (m, H-7), 7.75 (d, *J =* 7.8 Hz, H-8), 12.17 (s, NH-9), 8.12 (d, *J =* 8.0 Hz, H-2'/6'), 7.68 (d, *J =* 8.0 Hz, H-3'/5'), 7.59–7.66 (m, H-4'), 7.54 (s, C=CH), 9.30 (s, H-2''), 8.34 (d, *J =* 7.5 Hz, H-4''), 7.85 (t, *J =* 7.5 Hz, H-5''), 8.80 (d, *J =* 7.5 Hz, H-6''). ^13^C-NMR (DMSO-*d_6_*): *δ* 143.1 (C-1), 137.2 (C-3), 116.9 (C-4), 129.1 (C-4a), 120.9 (C-4b), 122 (C-5), 120.8 (C-6), 129.3 (C-7), 113.0 (C-8), 141.5 (C-8a), 134.8 (C-9a), 132.3 (C-1'), 128.6 (C-2'/6'), 128.9 (C-3'/5'), 129.0 (C-4'), 167.0 (C=O), 164.4 (C=N), 135.7 (C=CH), 126.8 (C=CH), 135.1 (C-1''), 126.2 (C-2''), 148.2 (C-3''), 124.8 (C-4''), 130.4 (C-5''), 137.8 (C-6''). EIMS, 70 eV, *m/z* (rel. int. %): 460 (30, M^+^), 243 (100), 271 (30), 432 (5). HR-ESIMS: calcd for C_27_H_17_N_4_O_4_: 461.1250 [M+H]^+^; found: 461.1296.

*2-[1-(4-Acetoxyphenyl)-9H-β-carbolin-3-yl]-4-(3-nitrobenzylidene)-4H-oxazol-5-one* (**9**). Yield: 30%, m.p. 255–257 °C. IR (KBr) ν_max_ cm^−1^: 1816 cm^−1^ (C=O), 1655 (C=N), 1553–1528 (C=C). ^1^H-NMR (DMSO-*d_6_*): *δ* 9.20 (s, H-4), 8.51 (d, *J =* 8.1 Hz, H-5), 7.41 (t, *J =* 8.1 Hz, H-6), 7.67 (d, *J =* 8.1 Hz, H-7), 7.75 (d, *J =* 8.1 Hz, H-8), 12.26 (s, NH-9), 8.20 (d, *J =* 8.4 Hz, H-2'/6'), 7.45 (d, *J =* 8.4 Hz, H-3'/5'), 2.37 (s, CH_3_), 7.56 (s, C=CH), 9.32 (s, H-2''), 8.34 (dd, *J =* 7.8 Hz and 2.1 Hz, H-4'), 7.87 (t, *J =* 7.8 Hz, H-5''), 8.83 (d, *J =* 7.8 Hz, H-6''). ^13^C-NMR (DMSO-*d_6_*): *δ* 142.3 (C-1), 141.5 (C-3), 117.0 (C-4), 129.1 (C-4a), 120.9 (C-4b), 122.1 (C-5), 121.0 (C-6), 129.4 (C-7), 113.0 (C-8), 144.1 (C-8a), 134.7 (C-9a), 132.3 (C-1'), 129.8 (C-2'/6'), 122.3 (C-3'/5'), 151.3 (C-4'), 20.9 (CH_3_), 169.2 (OCOCH_3_), 166.9 (C=O), 164.4 (C=N), 135.7 (C=CH), 126.4 (C=CH), 135.1 (C-1''), 126.2 (C-2''), 148.2 (C-3''), 124.8 (C-4''), 130.4 (C-5''), 137.8 (C-6''). HR-ESIMS: calcd for C_29_H_19_N_4_O_6_ [M+H]^+^: 519.1305; found: 519.1400.

*2-[1-(4-Methoxyphenyl)-9H-β-carbolin-3-yl]-4-(3-nitrobenzylidene)-4H-oxazol-5-one* (**10**). Yield: 32%, m.p. 201–203 °C. IR (KBr) ν_max_ cm^−1^: 3330 (N–H), 1781 cm^−1^ (C=O), 1609 (C=N), 1547–1494 (C=C). ^1^H-NMR (DMSO-*d_6_*): *δ* 9.15 (s, H-4), 8.48 (d, *J =* 7.6 Hz, H-5), 7.40 (t, *J =* 7.6 Hz, H-6), 7.65 (t, *J =* 7.6 Hz, H-7), 7.71 (d, *J =* 7.6 Hz, H-8), 12.14 (s, NH-9), 8.12 (d, *J =* 8.7 Hz, H-2'/6'), 7.24 (d, *J =* 8.7 Hz, H-3'/5'), 3.91 (s, OCH_3_), 7.55 (s, C=CH), 9.32 (s, H-2''), 8.35 (d, *J =* 8.0 Hz, H-4''), 7.87 (t, *J =* 8.0 Hz, H-5”), 8.51 (d, *J =* 8.0 Hz, H-6''). ^13^C-NMR (DMSO-*d_6_*): *δ* 143.1 (C-1), 138.0 (C-3), 116.6 (C-4), 129.2 (C-4a), 120.0 (C-4b), 122.0 (C-5), 121.0 (C-6), 128.9 (C-7), 109.9 (C-8), 142.0 (C-8a), 134.7 (C-9a), 129.6 (C-1'), 130.1 (C-2'/6'), 114.3(C-3'/5'), 160.2 (C-4'), 55.4 (OCH_3_), 167.1 (C=O), 164.5 (C=N), 135.8 (C=CH), 126.7 (C=CH), 135.2(C-1''), 126.2 (C-2''), 148.2 (C-3''), 124.8 (C-4''), 131.0 (C-5''), 137.8 (C-6''). EIMS, 70 eV, *m/z* (rel. int. %): 490 (60, M^+∙^), 301 (30), 273 (100), 258 (35). HR-ESIMS: calcd for C_28_H_19_N_4_O_5_ [M+H]^+^: 491.1355; found: 491.1309. 

*2-[1-(4-Methoxyphenyl)-9H-β-carbolin-3-yl]-4-benzylidene-4H-oxazol-5-one* (**11**). Yield: 40%, m.p. 245–247 °C. IR (KBr) ν_max_ cm^−1^: 3330 (N–H), 1781 cm^−1^ (C=O), 1648 (C=N), 1573–1494 (C=C). ^1^H-NMR (DMSO-*d_6_*): *δ* 9.18 (s, H-4), 8.55 (d, *J =* 7.5 Hz, H-5), 7.41 (m, H-6), 7.64 (t, *J =* 7.5 Hz, H-7), 7.74 (*d*, *J =* 7.5 Hz, H-8), 12.00 (s, NH-9), 8.06 (d, *J =* 8.4 Hz, H- 2'/6'), 7.24 (d, *J =* 8.4 Hz, H-3'/5'), 3.90 (s, OCH_3_), 7.56 (s, C=CH), 8.40 (d, *J =* 7.2 Hz, H-2''/6''), 7.58 (d, *J =* 7.2 Hz, H-3''/H-5''), 7.37 (m, H-4''). ^13^C-NMR (DMSO-*d_6_*): *δ* 143.4 (C-1), 138.2 (C-3), 116.7 (C-4), 129.6 (C-4a) 121.5 (C-4b), 122.7 (C-5), 121.1 (C-6), 129.4 (C-7), 113.4 (C-8), 141.9 (C-8a), 135 (C-9a), 133.1 (C-1'), 130.5 (C-2'/6'), 114.7 (C-3'/5'), 160.6 (C_0_-4'), 55.5 (OCH_3_), 168.0 (C=O), 164.0 (C=N), 134.1 (C=CH), 131.5 (C=CH), 134 (C-1''), 132.7 (C-2''/6), 129.4 (C-3''/5''), 130.0 (C-4''). EIMS, 70 eV, *m/z* (rel. int. %): 445 (35, M^+^), 301 (30), 273 (100), 258 (35), 242 (15). HR-ESIMS: calcd for C_28_H_20_N_3_O_3_ [M+H]^+^: 446.1505; found: 446.1529.

### 3.5. Biological Assays

#### 3.5.1. Cytotoxic Assay

The synthesized compounds were evaluated *in vitro* against six human cancer cell lines consisting of glioma (U251), melanoma (UACC-62), breast (MCF-7), prostate (PC-3), ovarian (OVCAR-03) and colon (HT-29). Cell lines were obtained from National Cancer Institute (Frederick, MD, USA). Normal cell line (VERO, renal, green monkey), from the Rio de Janeiro Cell Bank, was also used. Stock cultures were grown in medium containing 5 mL RPMI 1640 (GIBCO BRL) supplemented with 5% fetal bovine serum (FBS, GIBCO), at 37 °C with 5% CO_2_. Penicillin:streptomycin (1000 μg/L:1000 U/L, 1 mL/L) was added to the experimental cultures. Cells in 96-well plates (100 μL cells well^−1^) were exposed to compounds **8–11** in DMSO (concentrations of 0.25, 2.5, 25, and 250 μg·mL^−1^) at 37 °C, 5% of CO_2_ in air for 48 h. Final DMSO concentration did not affect the cell viability. Afterwards cells were fixed with 50% trichloroacetic acid and cell proliferation determined by spectrophotometric quantification (540 nm) of cellular protein content by using sulforhodamine B assay and doxorubicin as the positive control [36]. Three measurements were obtained: at time zero (To, at the beginning of incubation) and 48 h post-incubation for compound-free (C) and tested (T) cells. Cell proliferation was determined according to the equation 100 × [(T − T0)/C − T0], for T0 < T ≤ C, and 100 × [(T − T0)/T0], for T ≤ T0 and a concentration-response curve for each cell line was plotted using software ORIGIN 8.0^®^ (OriginLab Corporation). Using the concentration–response curve for each cell line, the IC_50_ values (concentration that produces a 50% reduction in cellular growth when compared to untreated control cells) and IC_100_ values (concentration that promotes total growth inhibition) were determined through non-linear regression analysis using software ORIGIN 8.0^®^ (OriginLab Corporation) [37]. Compounds with IC_50_ values < 100 μM were considered actives. 

#### 3.5.2. Antimicrobial Activity Assay

Antibacterial and antifungal assays for compounds **8–11** were carried out according to previously reported experimental protocols [38,39]. The bacterial strains were grown overnight at 36 °C in Nutrient Agar (Merck, Darmstadt, Germany), and the strains were grown in Saboraud Dextrose Agar. Inoculum for the assays was prepared by diluting scraped cell mass in 0.85% NaCl solution, adjusted to McFarland scale 0.5 and confirmed by spectrophotometrical reading at 580 nm. Cell suspensions were finally diluted to 10^4^ UFC·mL^−1^ for use in the activity assays. Minimal Inhibitory Concentration (MIC) tests were carried out using Müller-Hinton broth (bacteria) or RPMI-1640 (yeasts) on a tissue culture test plate (96 wells). Each compound was tested in duplicate. The stock solutions of compounds firstly in DMSO and subsequently in Tween 80 water solution (0.1%) were diluted and transferred into the first well, and serial dilutions were performed so that concentrations in the range of 250–1.6 μg·mL^−1^ were obtained. Chloramphenicol and nystatin (Merck) were used as the reference antibiotic control. The inoculum was added to all wells and the plates were incubated at 36 °C for 24 h. Antibacterial activity was detected by adding 0.5% aqueous solution of ntriphenyltetrazolium chloride (TTC, 20 μL, Merck). MIC was defined as the lowest concentration of the compounds that inhibited visible growth, as indicated by TTC staining (dead cells are not stained by TTC). For antifungal activity evaluation, after the incubation period changes in the RPMI-1640 medium color were verified from pink (original color) to yellow. The change indicates an acidification from medium by the microorganisms’ growth.

## 4. Conclusions

In conclusion, four 1-substituted β-carboline-3-(4-benzylidene)-*4H*-oxazol-5-ones **8–11** were prepared and assayed for their antitumor and antimicrobial activities. The compound 2-[1-(4-methoxyphenyl)-9H-β-carbolin-3-yl]-4-(benzylidene)*-4H-*oxazol-5-one (**11**) was the most active derivative, exhibiting potent cytotoxic activity against glioma (U251), prostate (PC-3) and ovarian (OVCAR-03) cancer cell lines with IC_50_ values of 0.48, 1.50 and 1.07 μM, respectively. *In silico* studies indicate that compounds of this class are potential new anticancer drug candidates.

## Supplementary Materials

Supplementary materials can be accessed at: http://www.mdpi.com/1420-3049/17/3/6099/s1.
