# Supplementary material for: Synthesis and Evaluation of New β-Carboline-3-(4-benzylidene)-4H-oxazol-5-one Derivatives as Antitumor Agents
Source: Molecules. 2012 May 21;17(5):6100–13. doi: 10.3390/molecules17056100 (PMC6268609; doi:10.3390/molecules17056100)

Molecules **2012**, *17*, 6100-6113; doi:10.3390/molecules17056100

OPEN ACCESS

molecules

ISSN 1420-3049

www.mdpi.com/journal/molecules

Article

Synthesis and Evaluation of New β-Carboline-3-(4-benzylidene)-*4H*-oxazol-5-one Derivatives as Antitumor Agents

Franciele Cristina Savariz [1], Mary Ann Foglio [2], João Ernesto de Carvalho [2], Ana Lúcia T. G. Ruiz [2], Marta C. T. Duarte [2], Mauricio Ferreira da Rosa [3], Emerson Meyer [1] and Maria Helena Sarragiotto [1,*]

[1] Departamento de Química, Centro de Ciências Exatas, Universidade Estadual de Maringá, Av. Colombo, 5790, Maringá, 87020-900 PR, Brazil

[2] Centro Pluridisciplinar de Pesquisas Químicas, Biológicas e Agrícolas (CPQBA), Universidade Estadual de Campinas, 6171, Campinas, 13083-970 SP, Brazil

[3] Centro de Engenharias e Ciências Exatas, Universidade Estadual do Oeste do Paraná, Rua da Faculdade, 645, Toledo, 85903-000 PR, Brazil

* Author to whom correspondence should be addressed; E-Mail: mhsarragiotto@uem.br; Tel.: +55-44-3261-3657; Fax: +55-44-3011-4125.

Received: 5 April 2012; in revised form: 5 May 2012 / Accepted: 7 May 2012 / Published: 21 May 2012

Supporting Information

Figure S1. ^{1}H-NMR spectra (300 MHz, DMSO-d_6) of compound **8**.

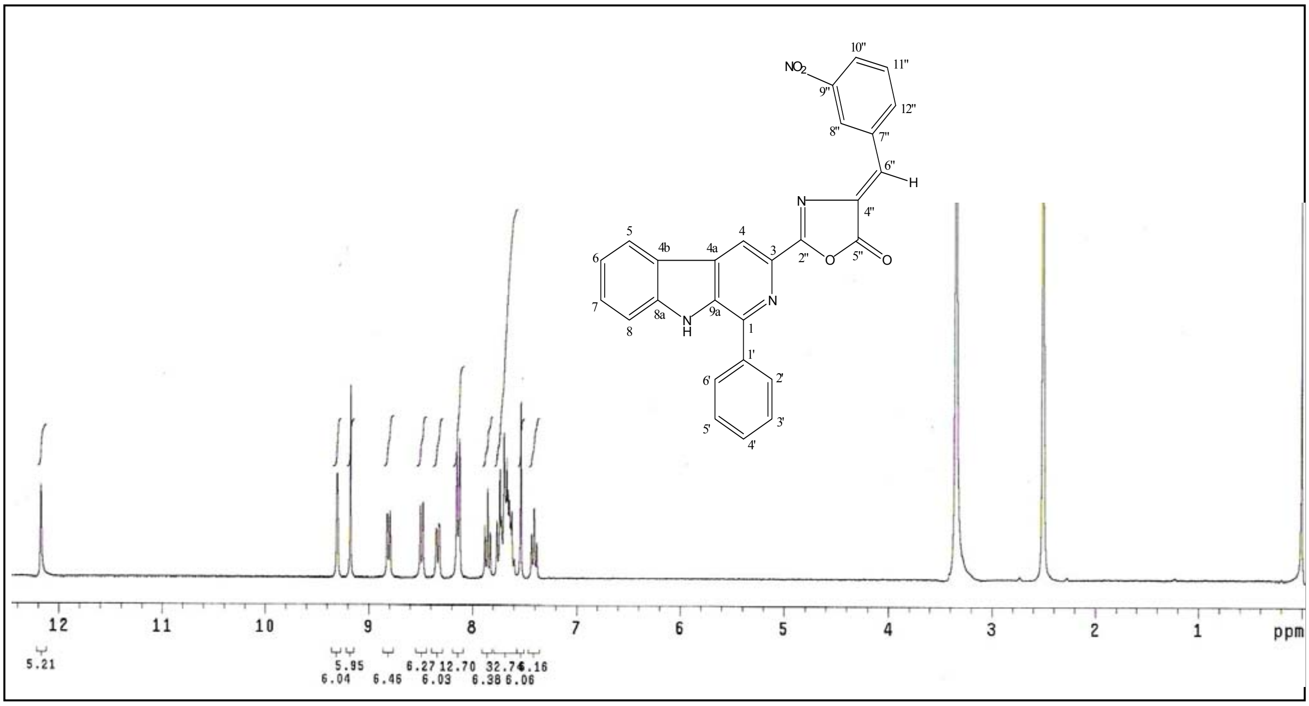

Figure S2. ^{13}C-NMR / DEPT spectra (75.5 MHz, DMSO-d_6) of compound **8**.

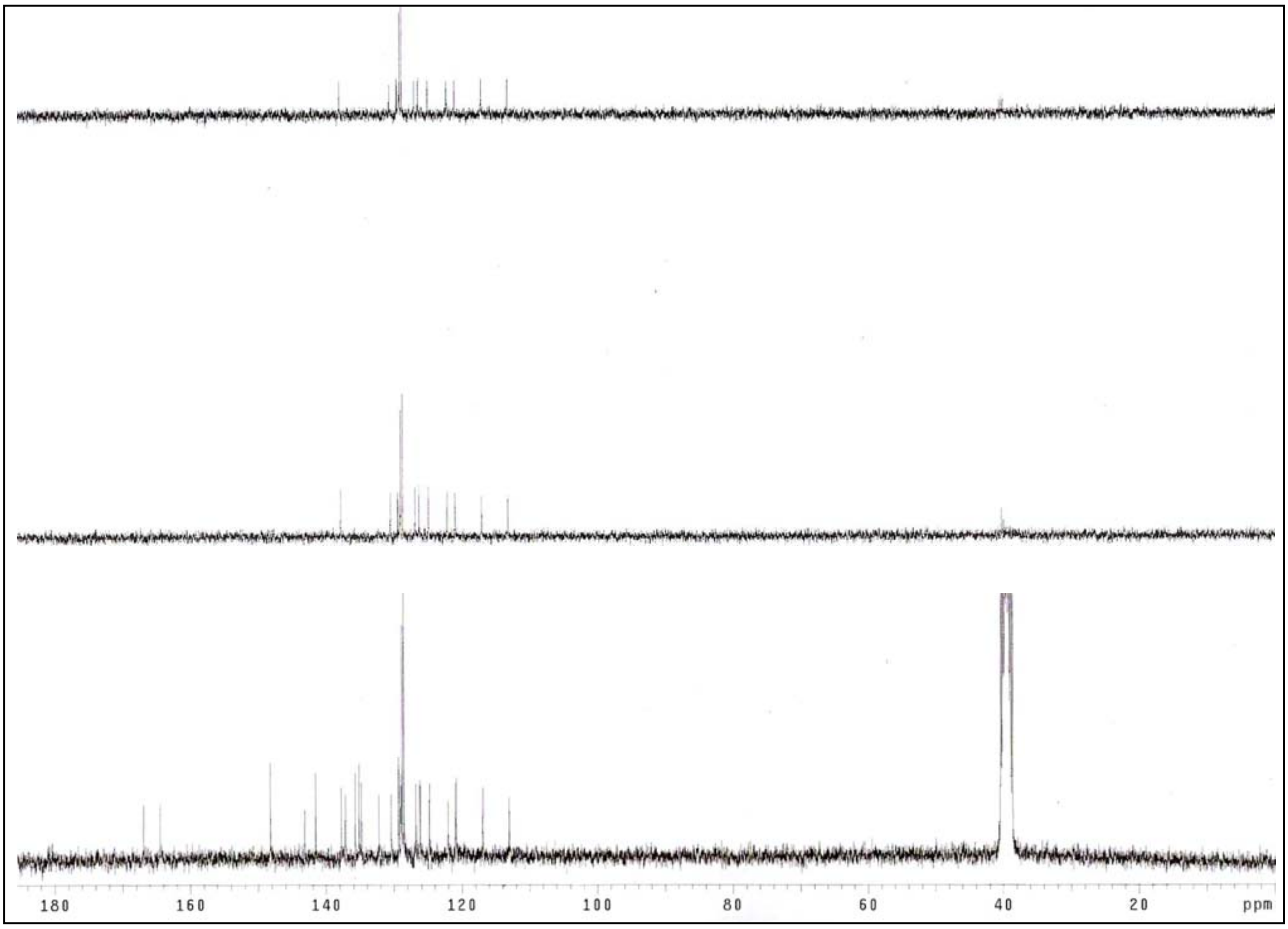

Figure S3. HSQC spectra (300 MHz/75.5 MHz, DMSO-d_6) of compound **8**.

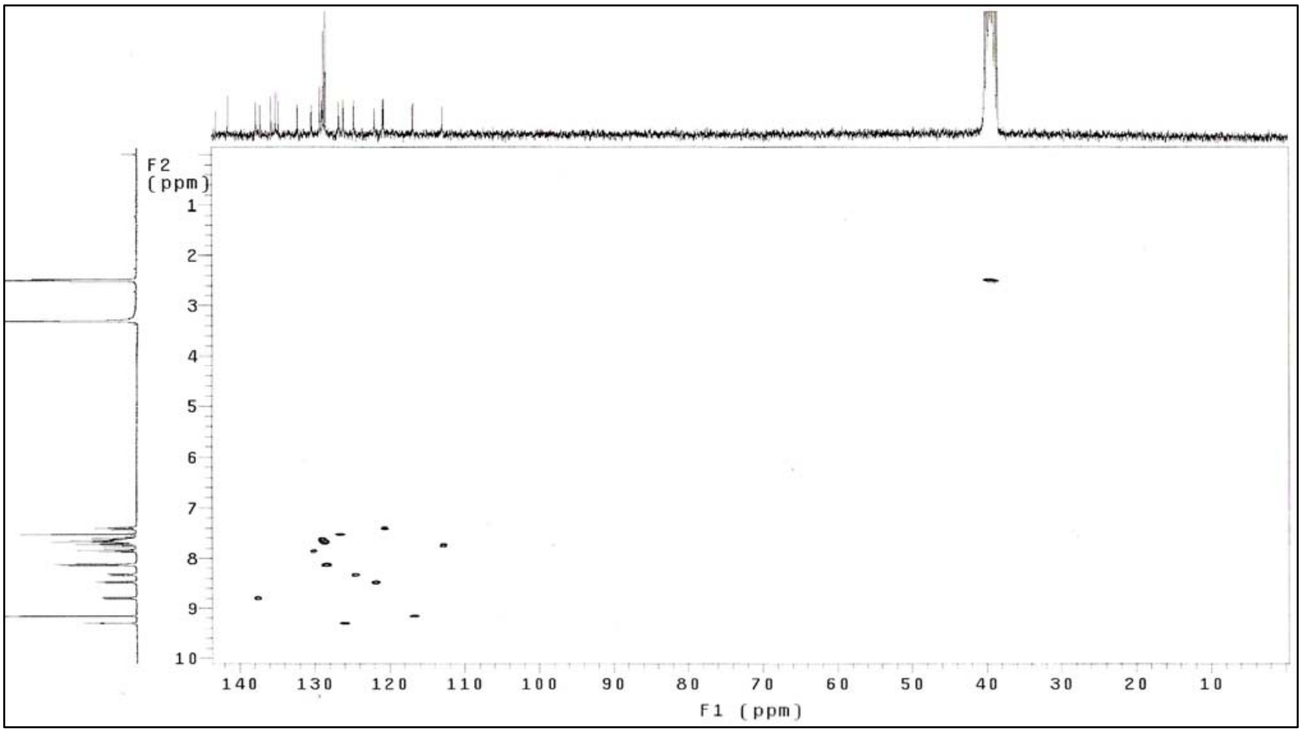

Figure S4. IR specta (KBr) of compound **8**.

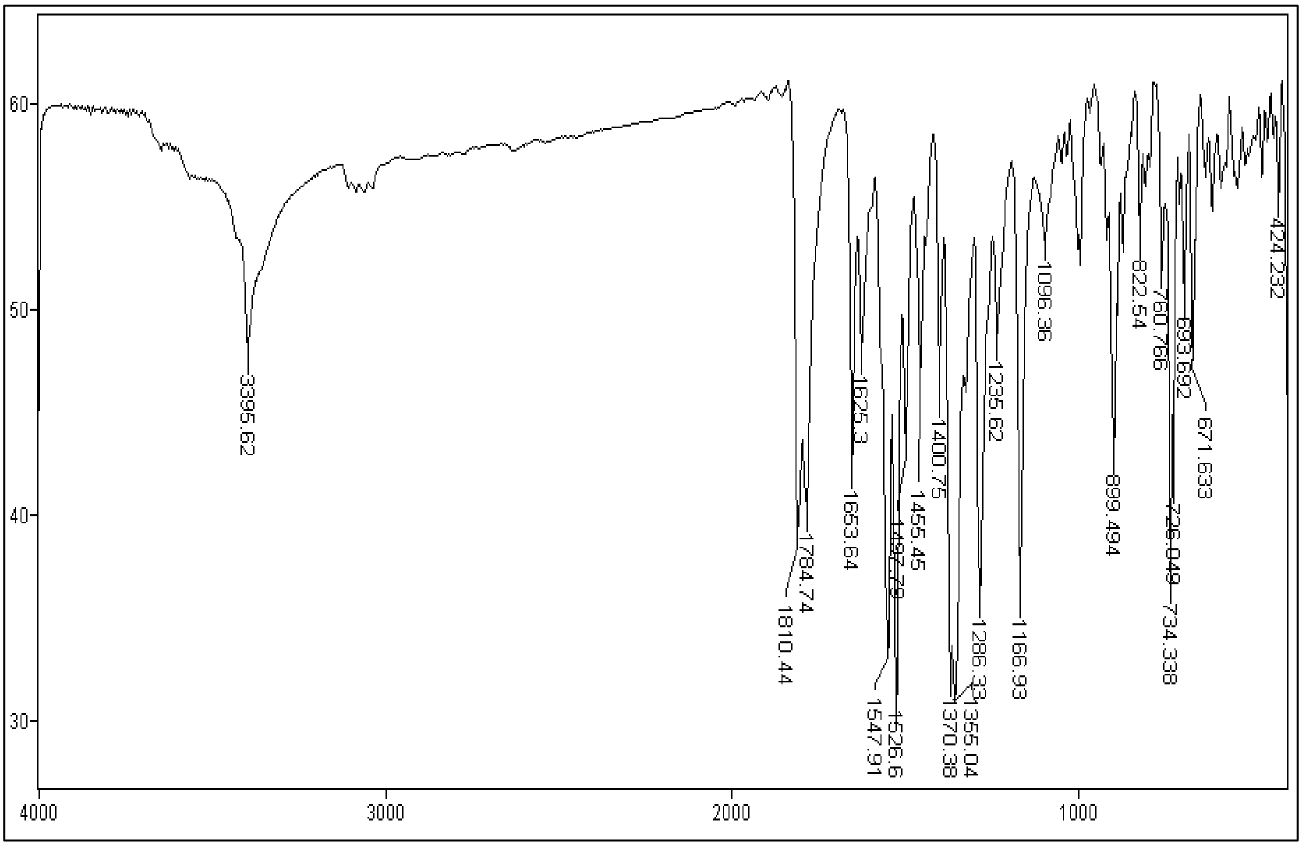

Figure S5. EI mass spectra (70 eV) of compound **8.**

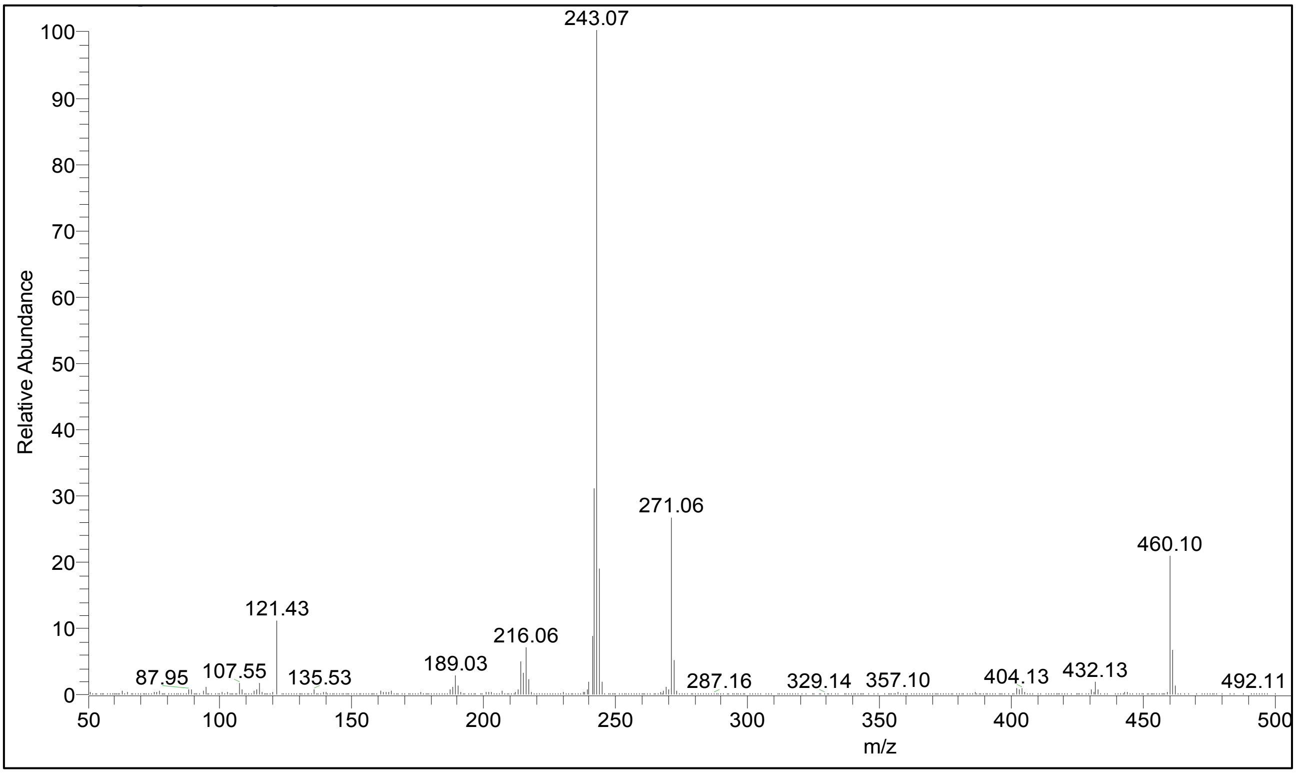

Figure S6. HR-ESI mass spectra of compound **8**.

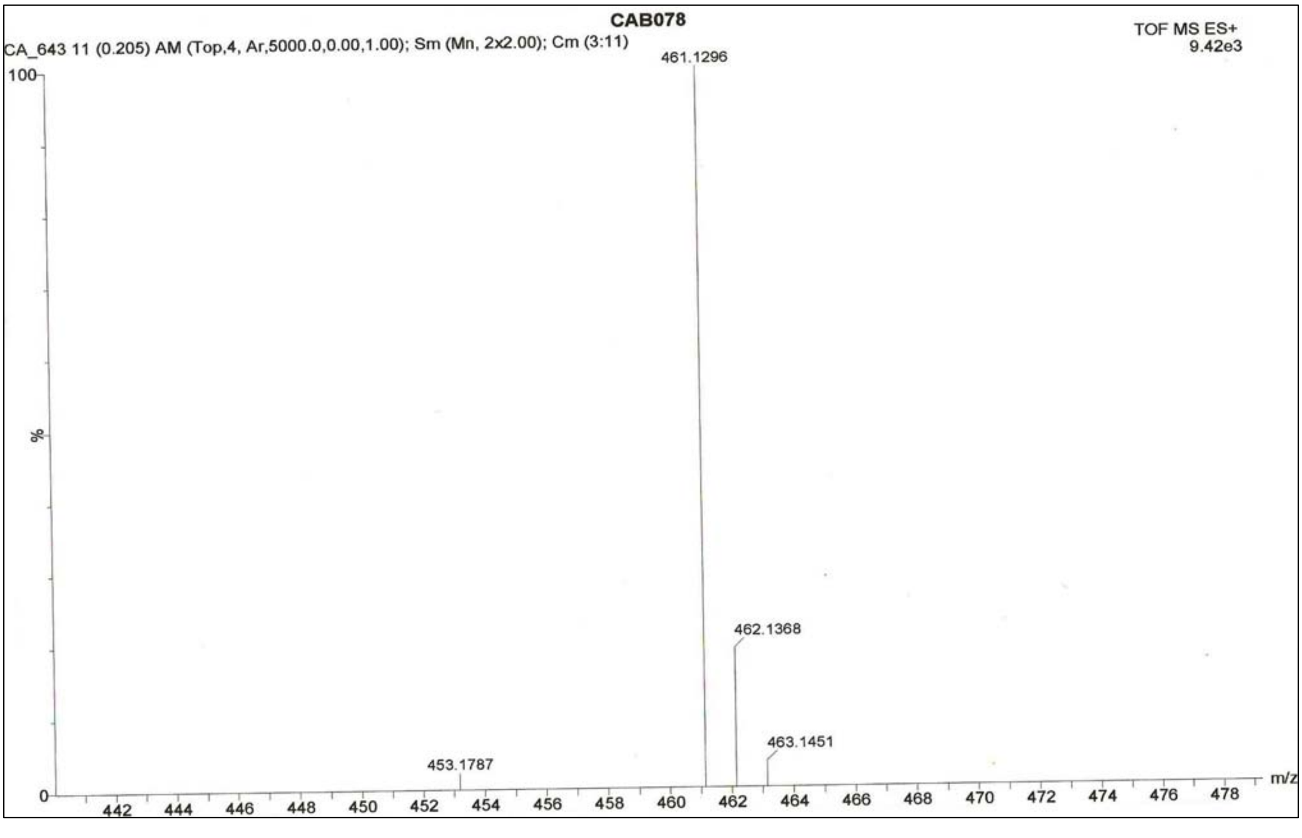

Figure S7. ^{1}H-NMR spectra (300 MHz, DMSO-d_6) of compound **9**.

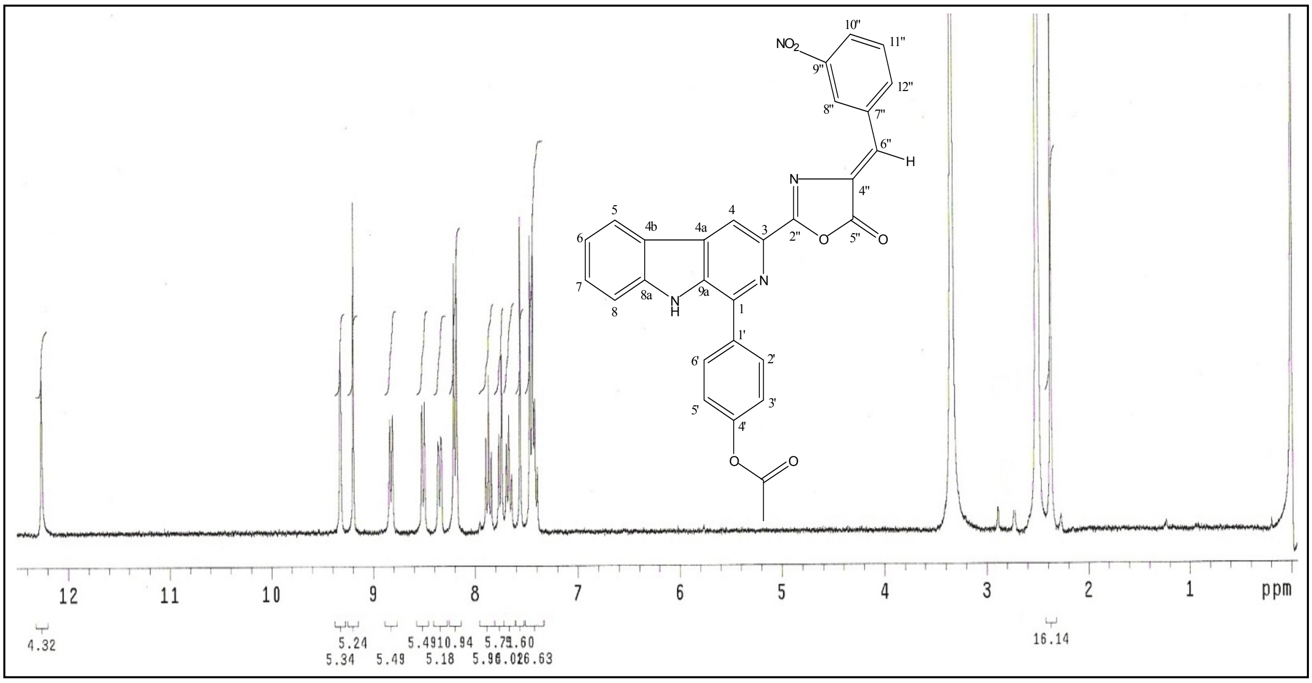

Figure S8. ^{13}C-NMR / DEPT spectra (75.5 MHz, DMSO-d_6) of compound **9**.

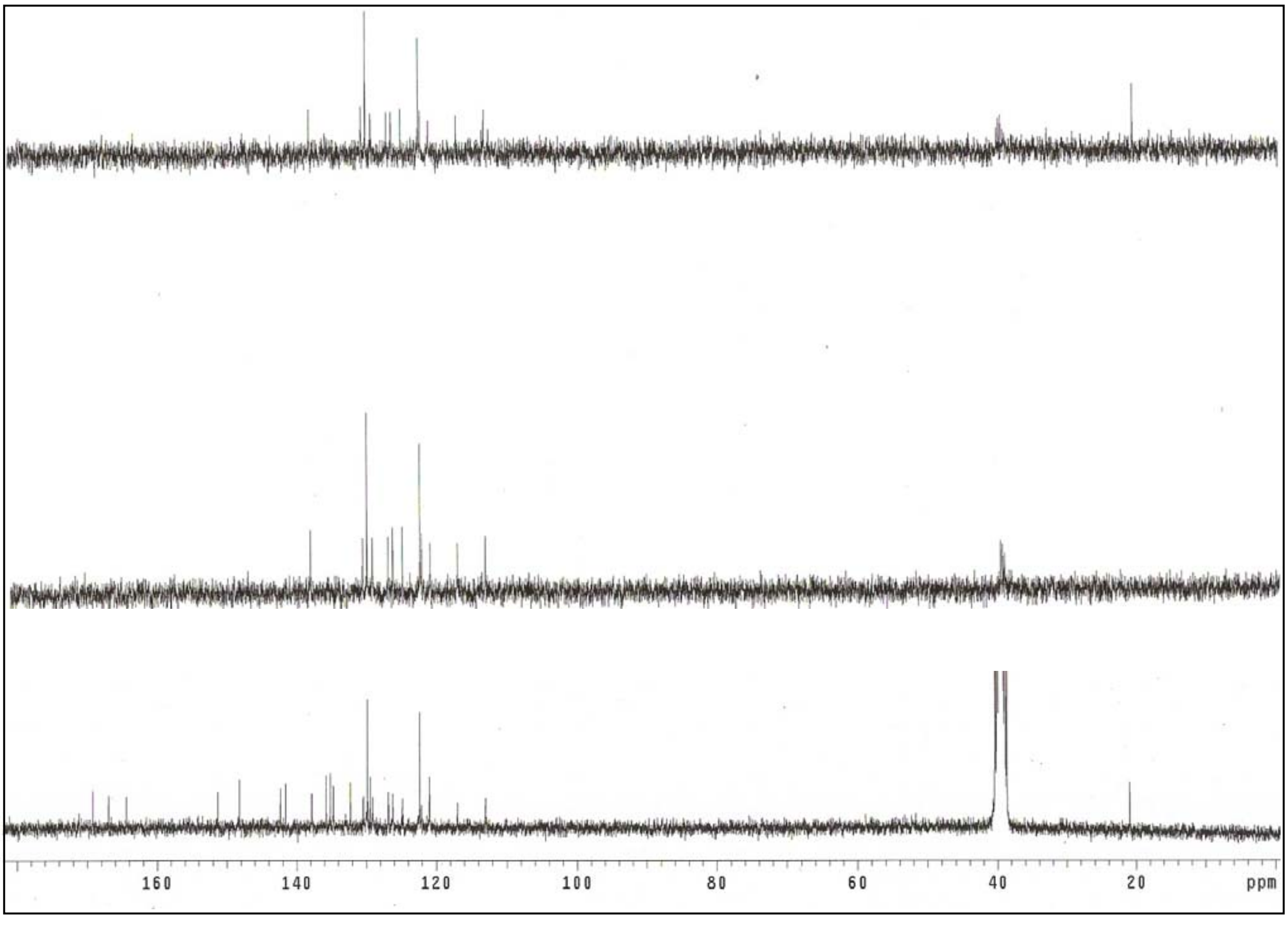

Figure S9. HSQC spectra (300 MHz/75.5 MHz, DMSO-d_6) of compound **9**.

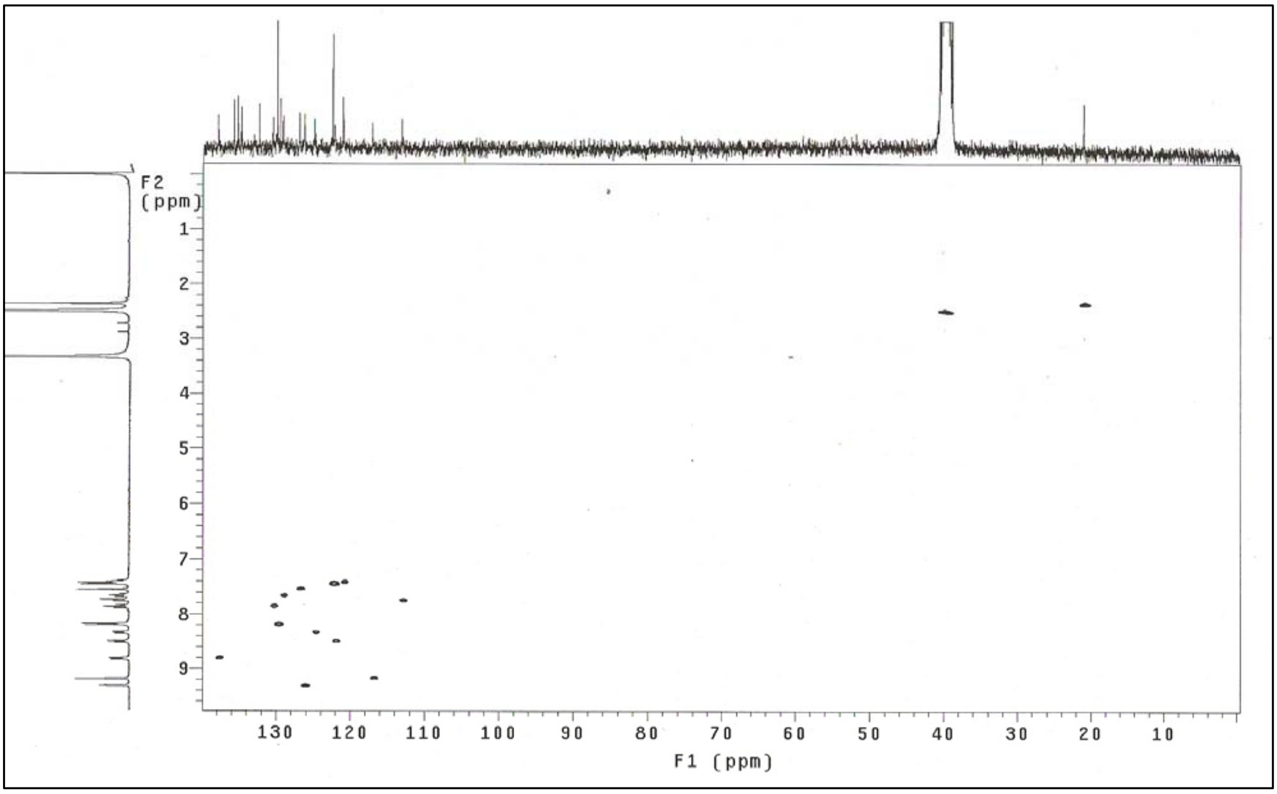

Figure S10. IR spectra (KBr) of compound **9**.

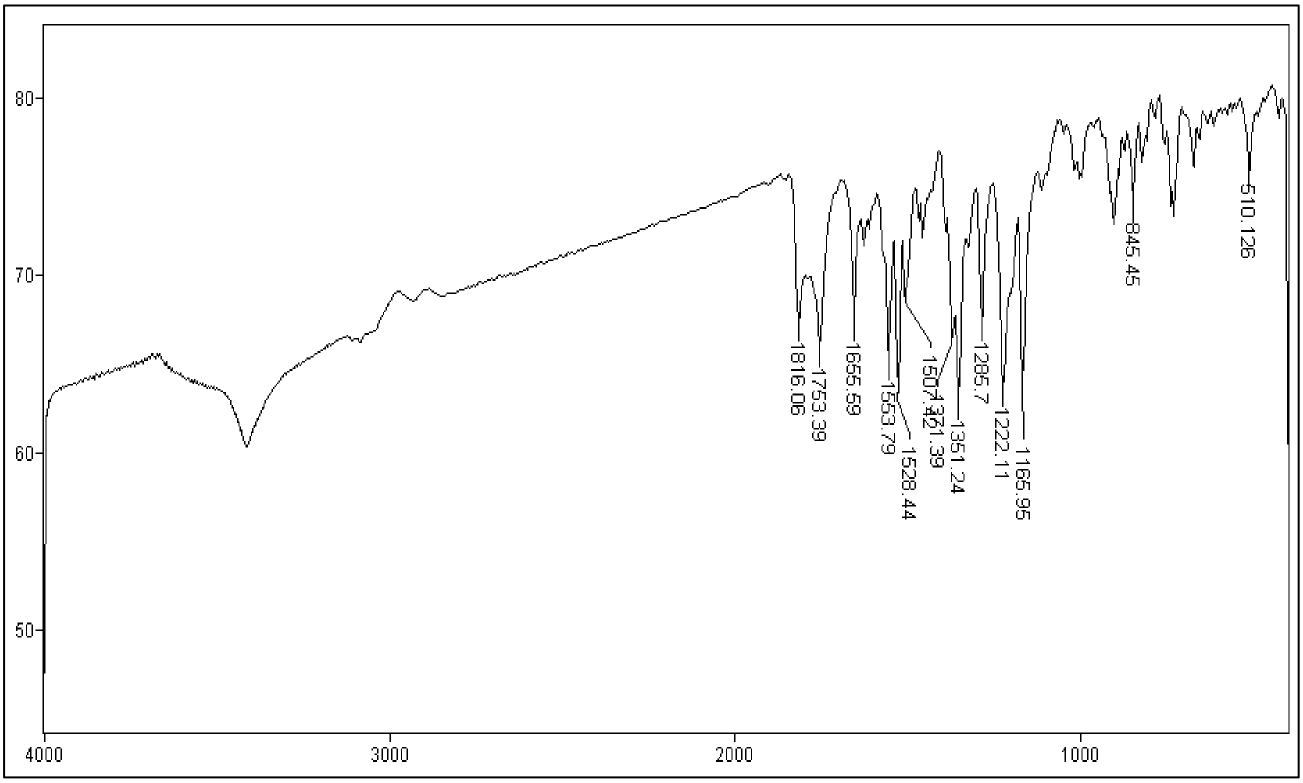

Figure S11. HR-ESI mass spectra of compound **9**.

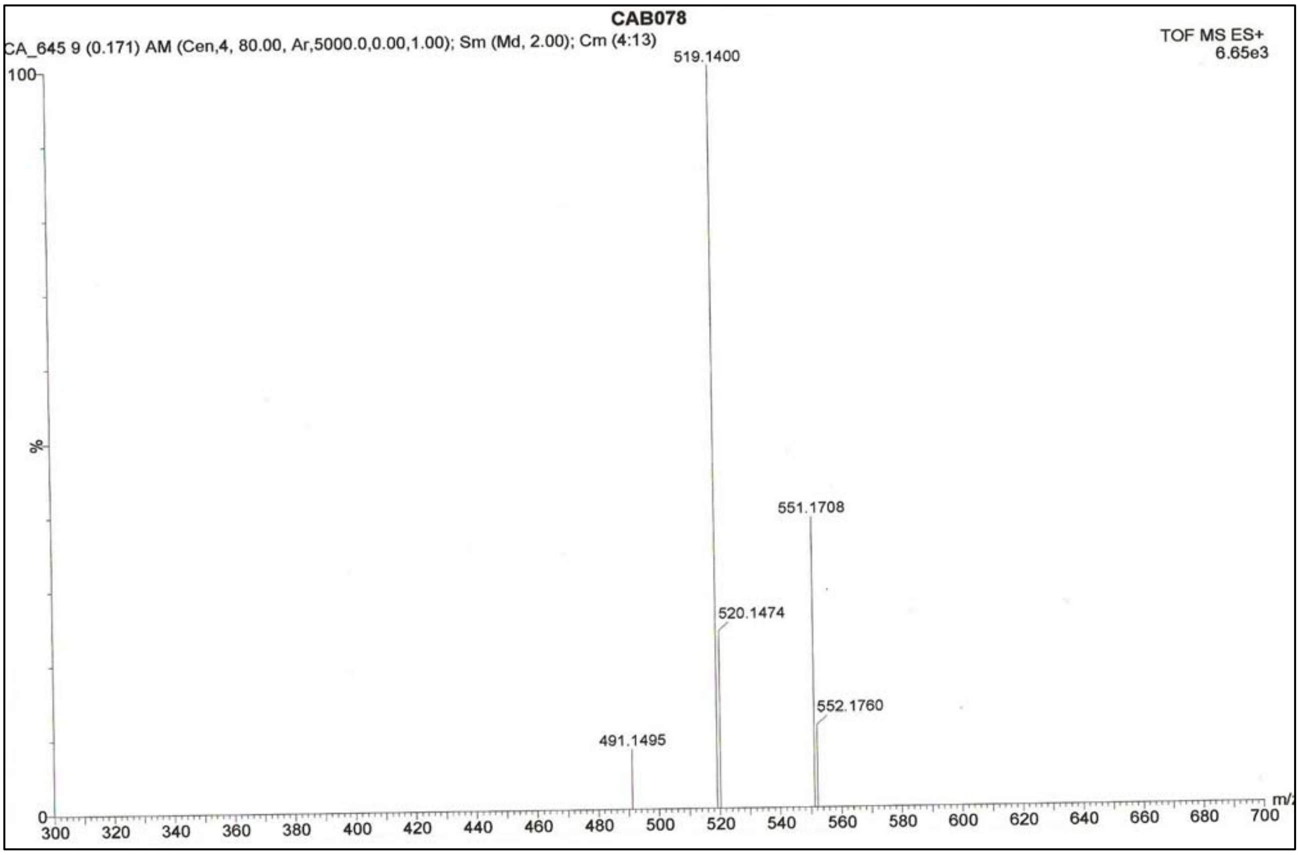

Figure S12. ^{1}H-NMR spectra (300 MHz, DMSO-d_6) of compound **10**.

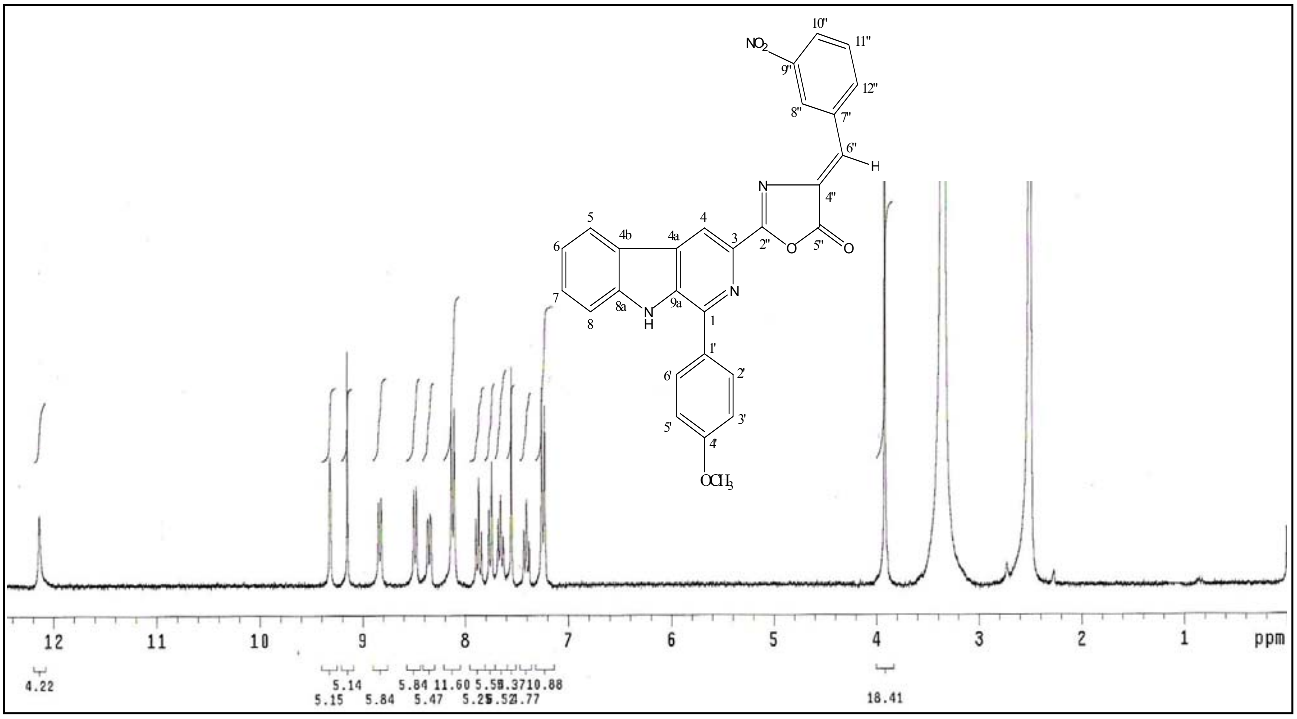

Figure S13. ^{13}C-NMR / DEPT spectra (75.5 MHz, DMSO-d_6) of compound **10**.

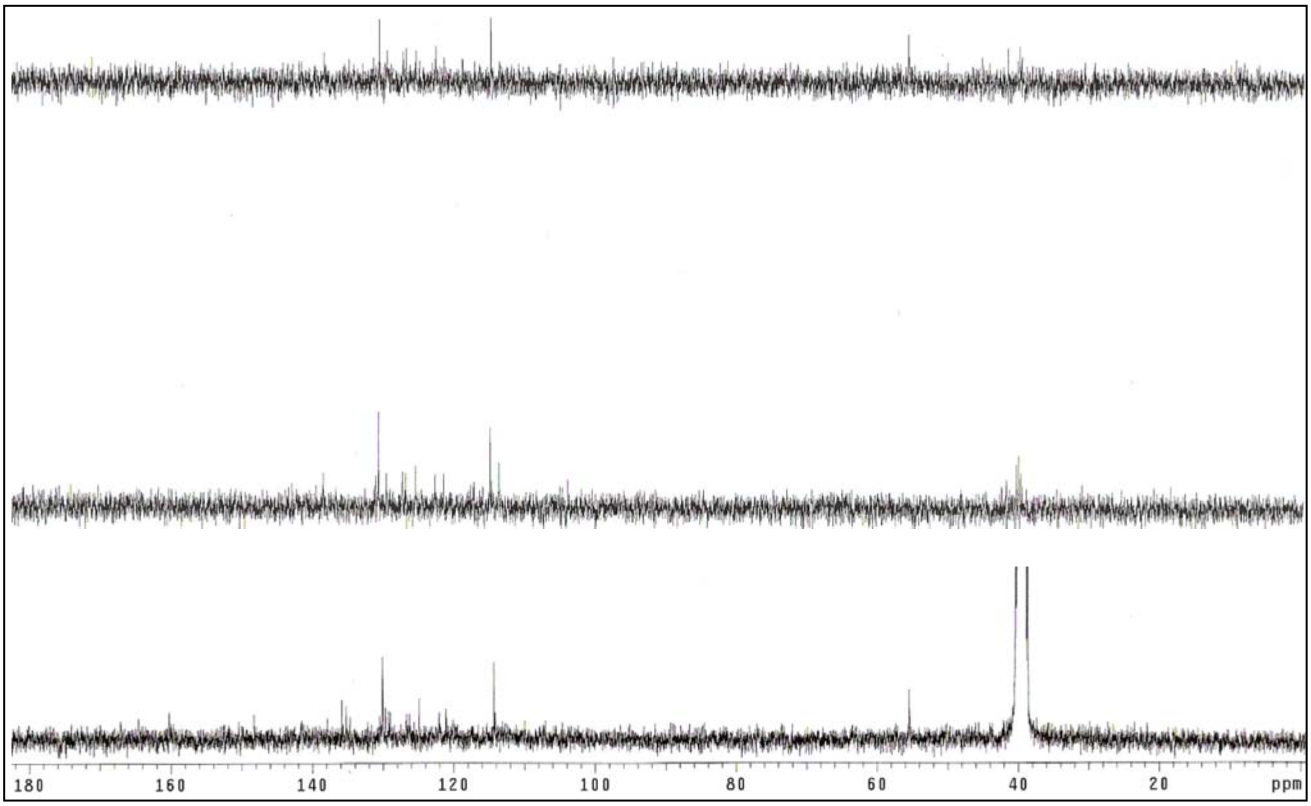

Figure S14. HSQC spectra (300 MHz/75.5 MHz, DMSO-d_6) of compound **10**.

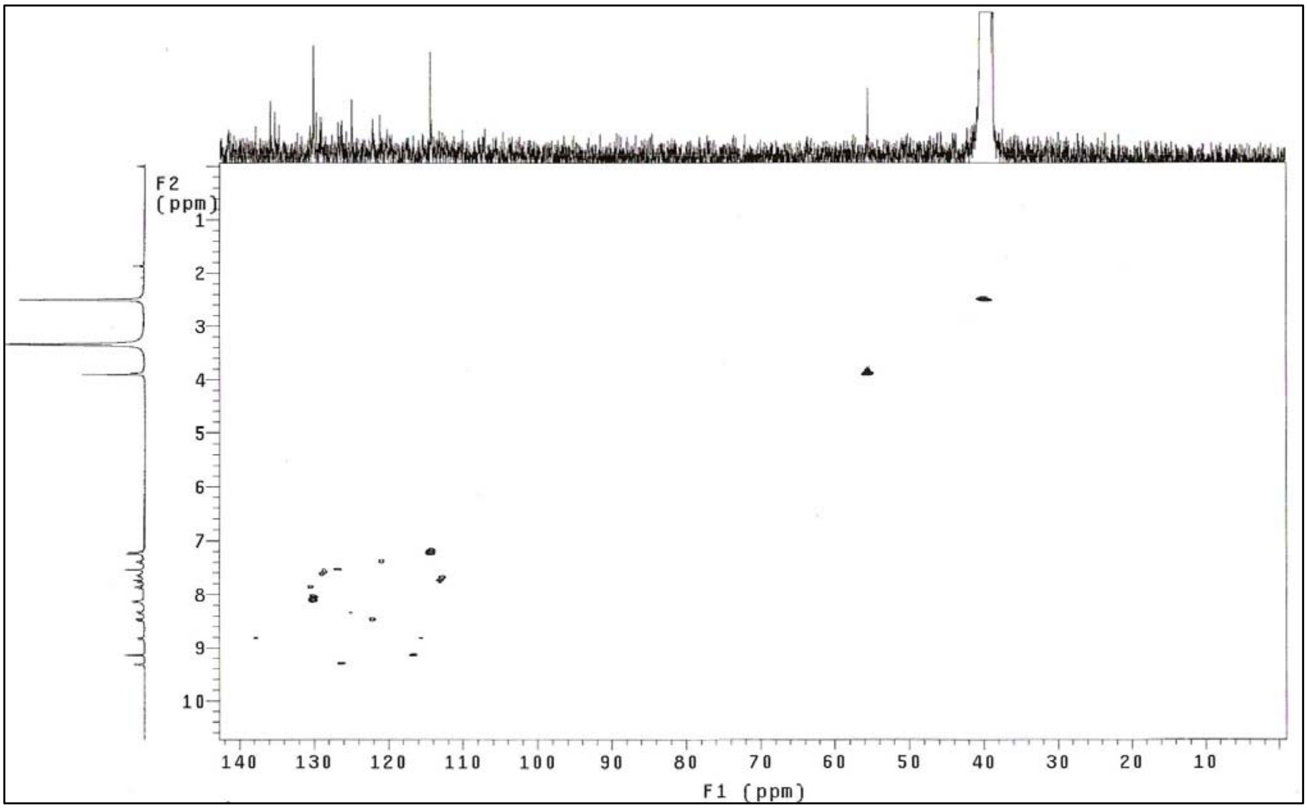

Figure S15. IR spectra (KBr) of compound **10**.

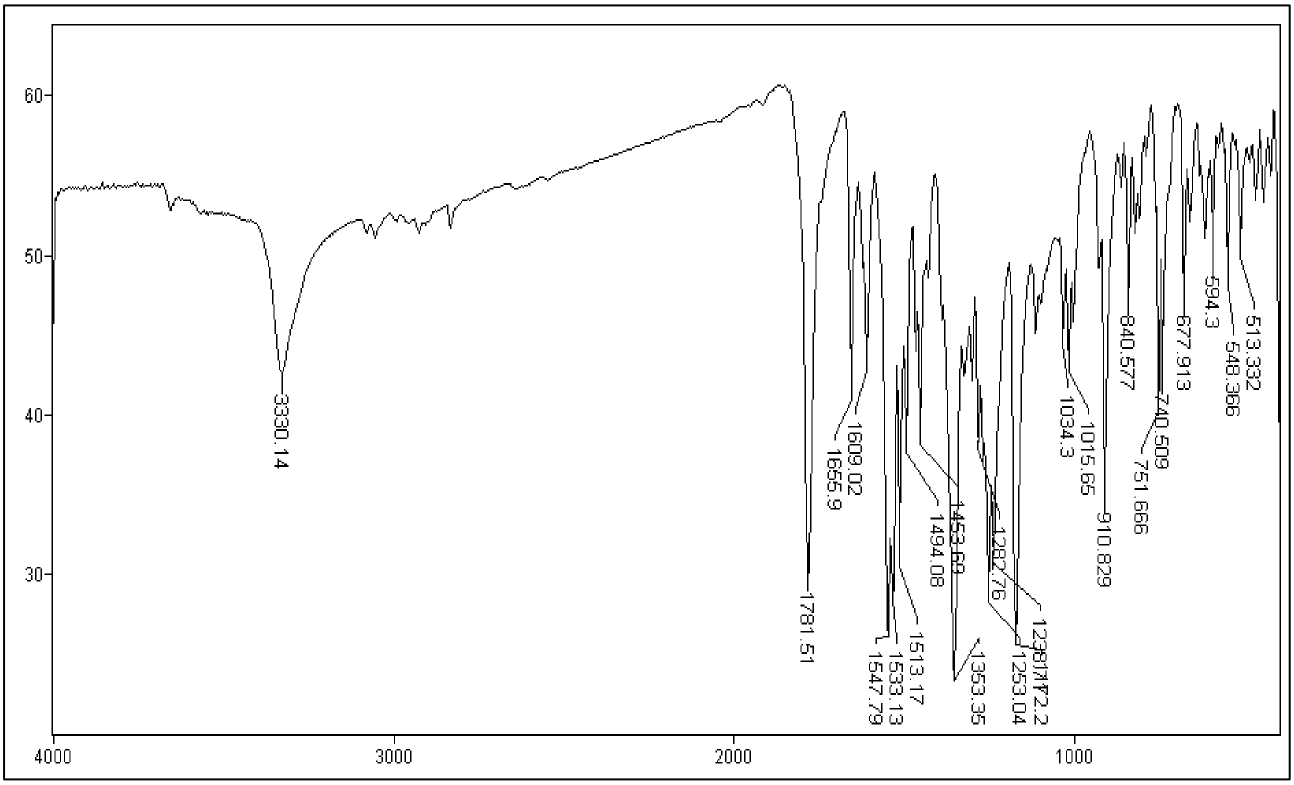

Figure S16. EI mass spectra (70 eV) of compound **10**.

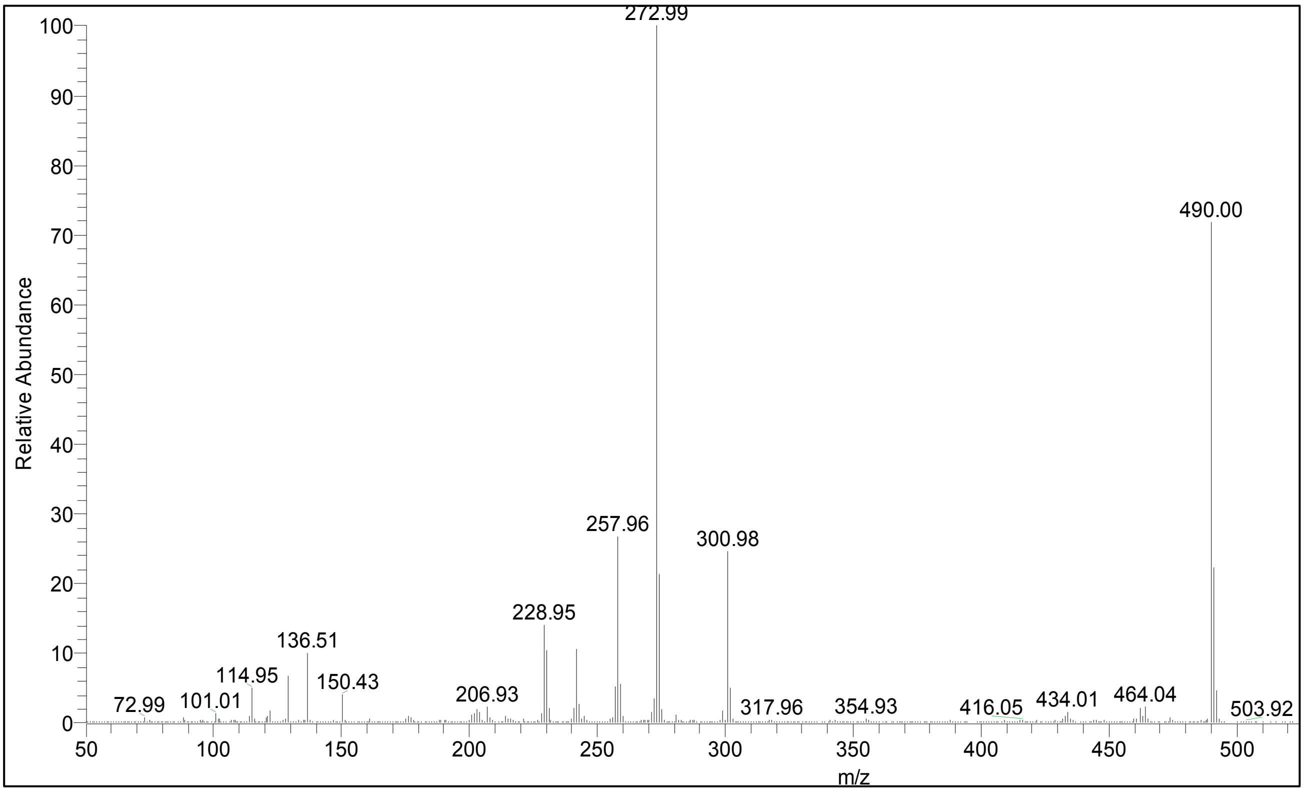

Figure S17. HR-ESI mass of compound **10**.

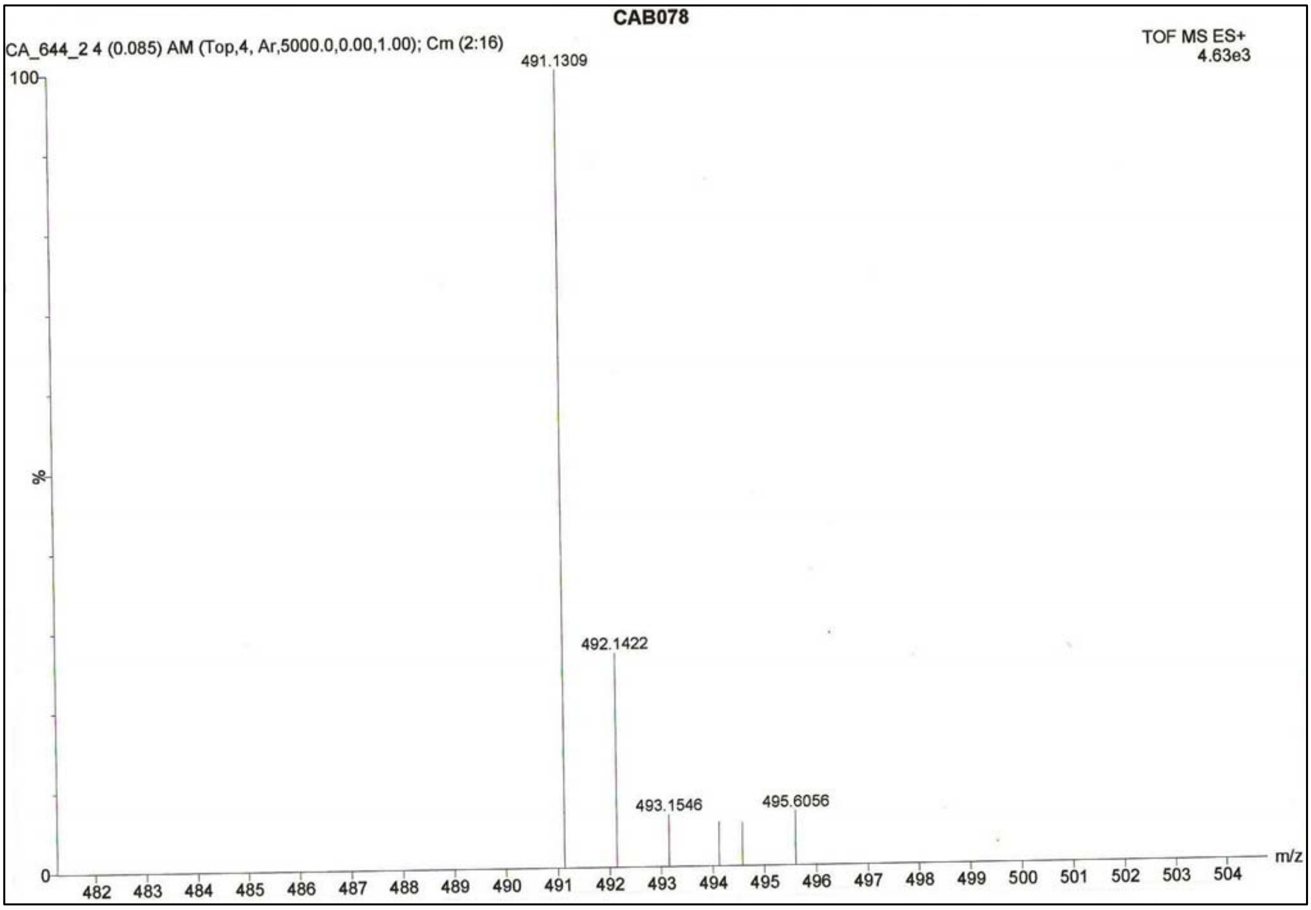

Figure S18. ^{1}H-NMR spectra (300 MHz, DMSO-d_6) of compound **11**.

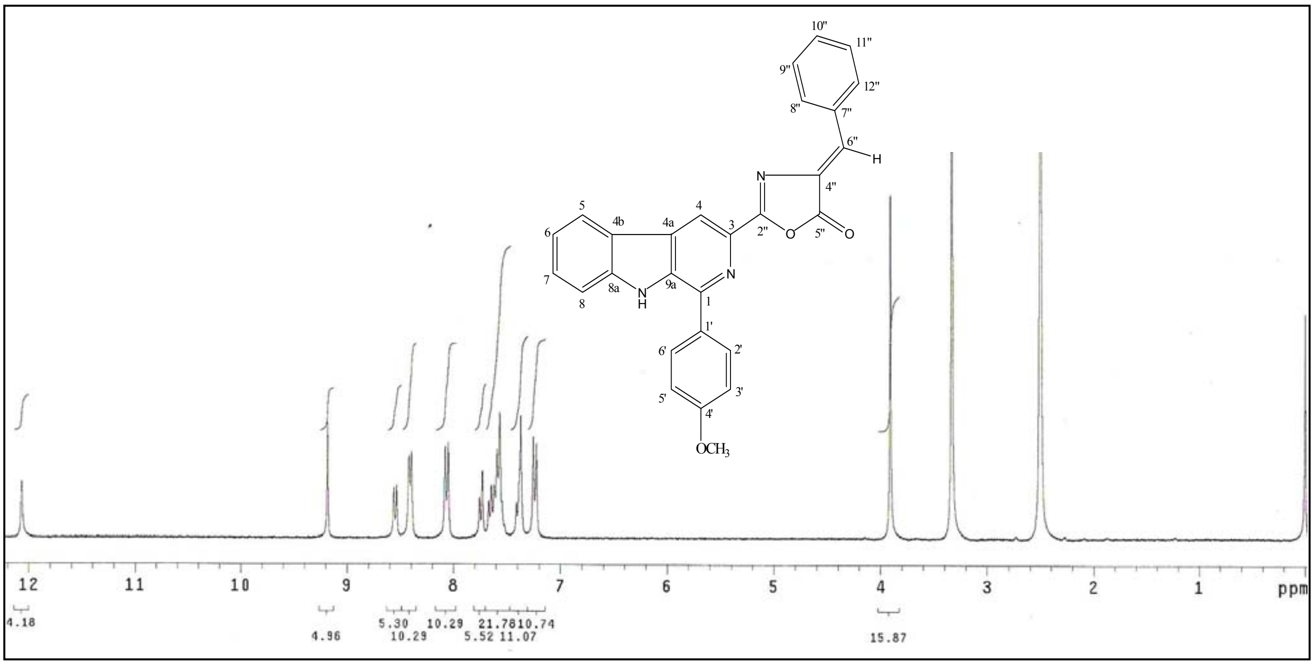

Figure S19. ^{13}C-NMR / DEPT spectra (75.5 MHz, DMSO-d_6) of compound **11**.

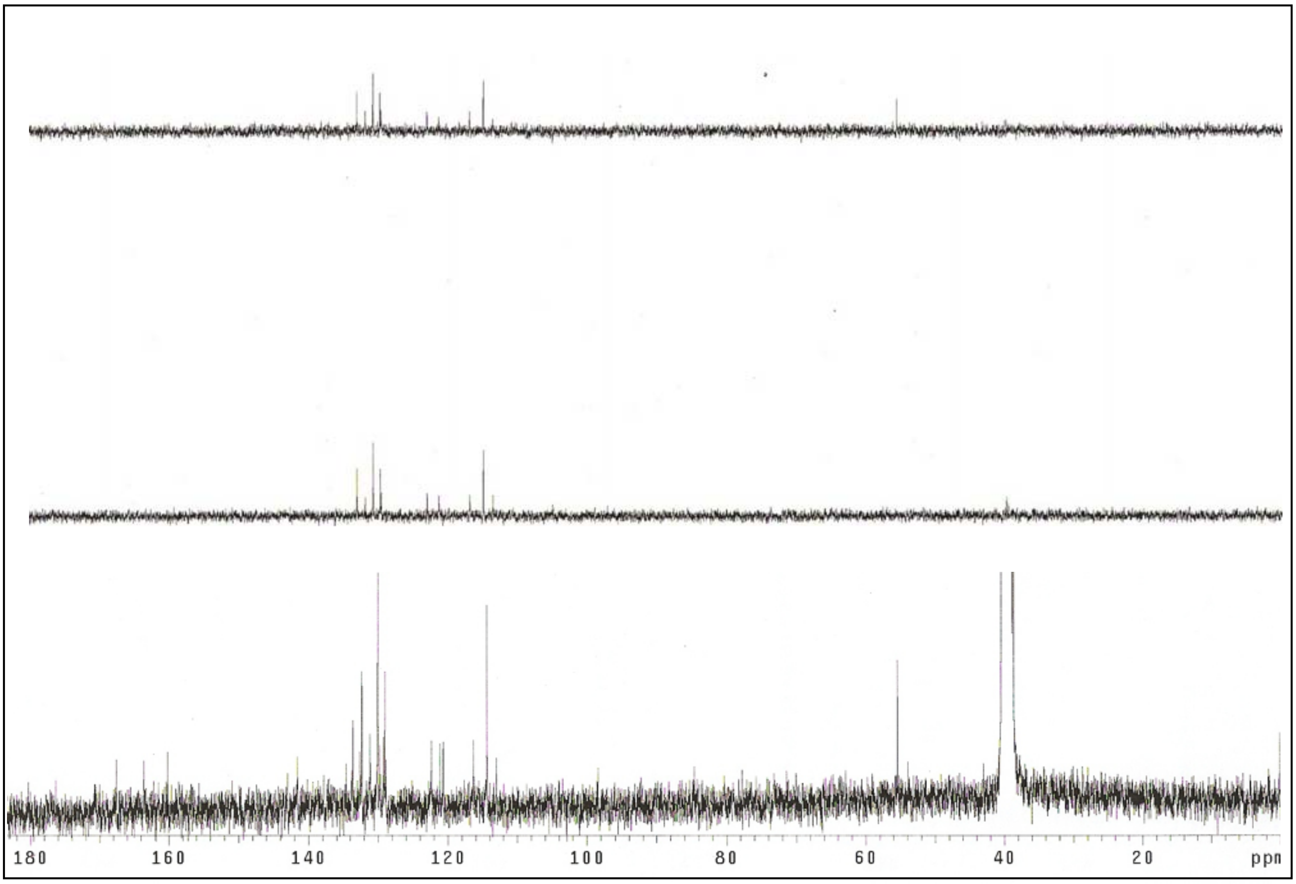

Figure S20. HSQC spectra (300 MHz/75.5 MHz, DMSO-d_6) of compound **11**.

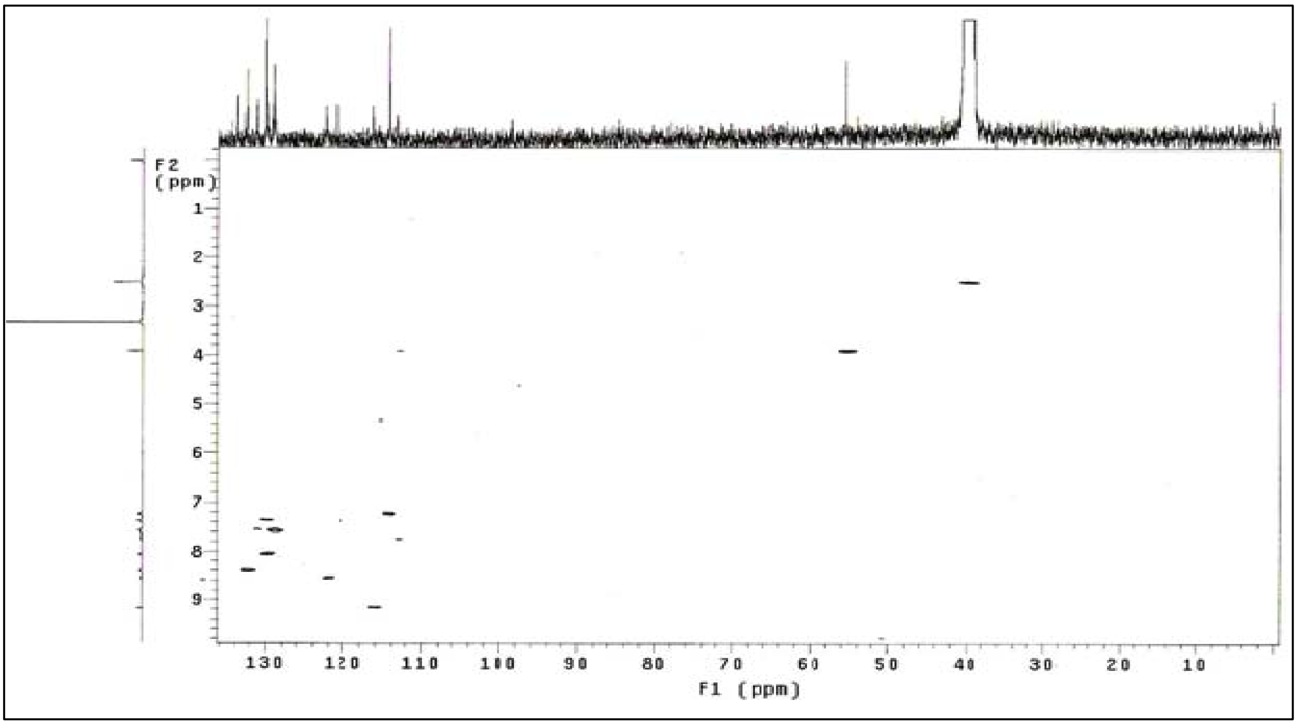

Figure S21. IR spectra (KBr) of compound **11**.

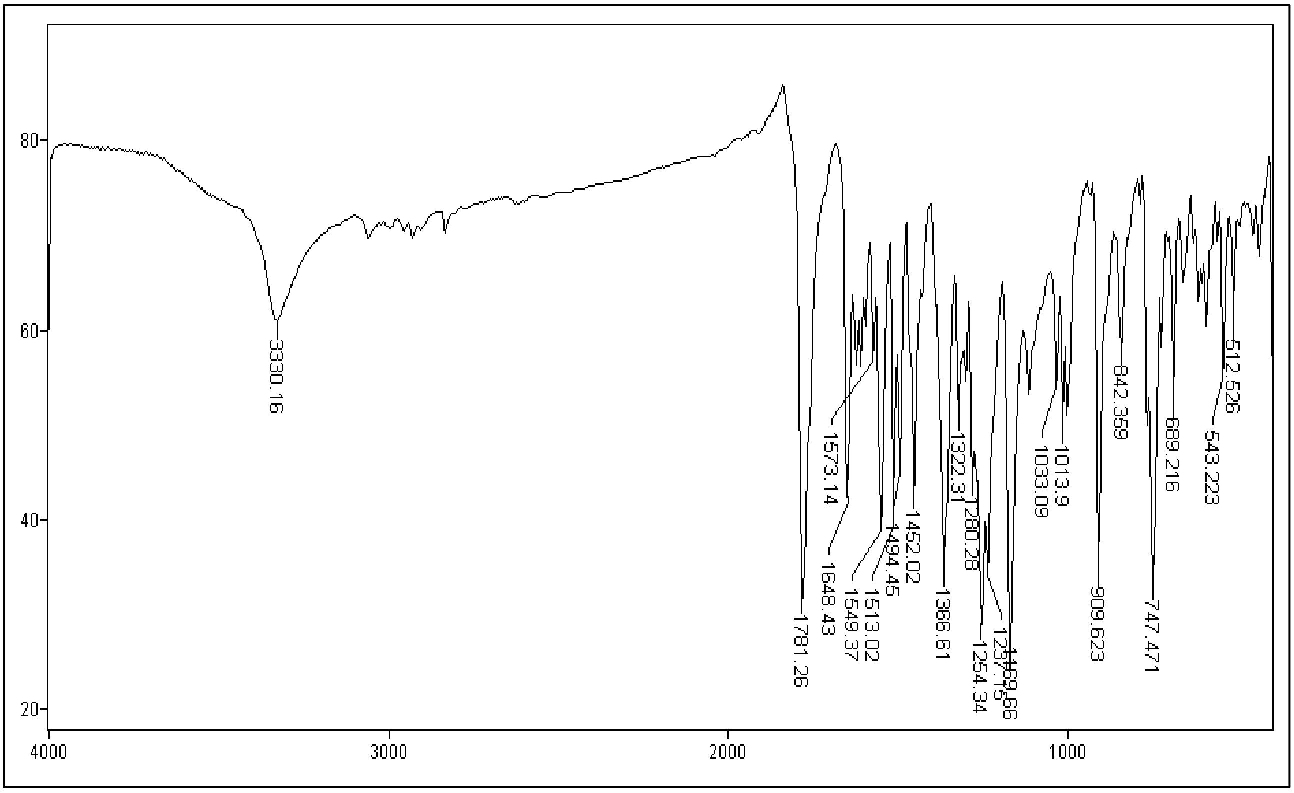

Figure S22. EI mass spectra (70eV) of compound **11**.

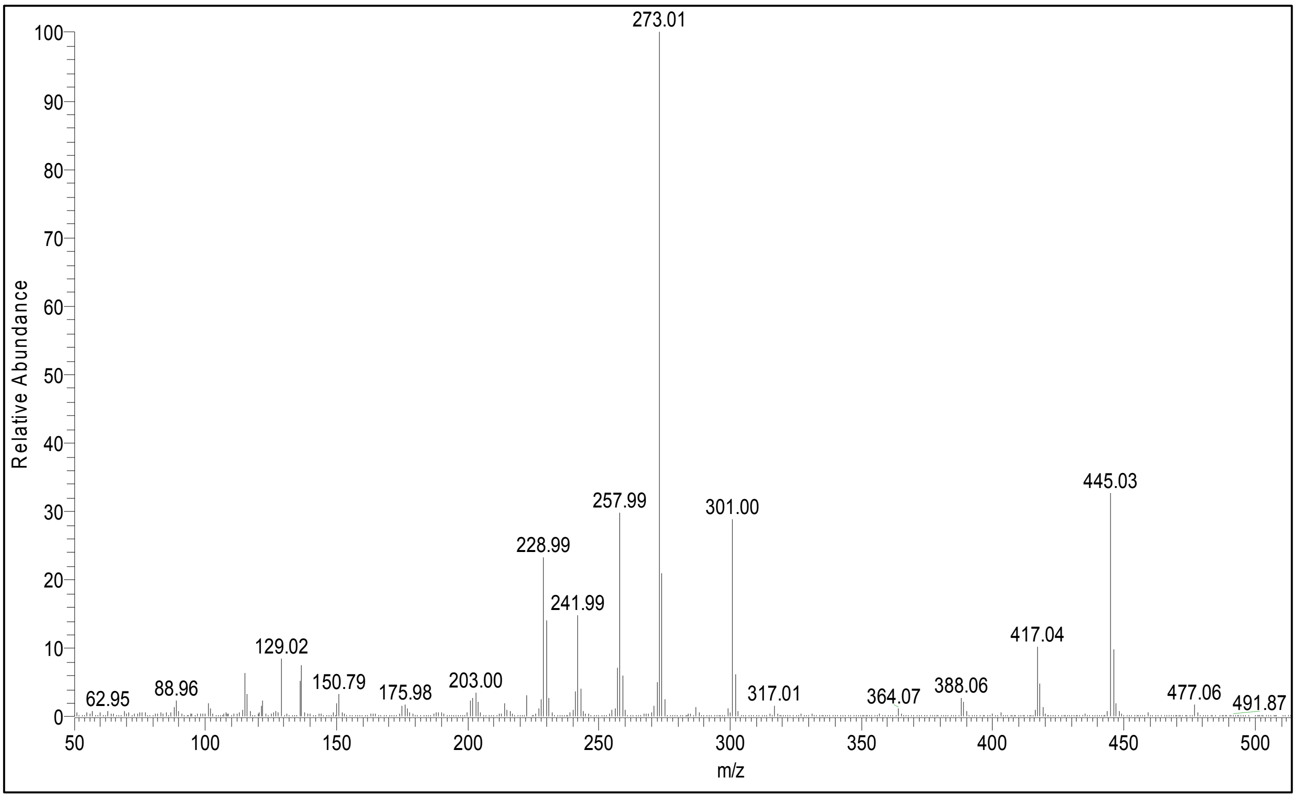

Figure S23. HR-ESI mass spectra of compound **11**.

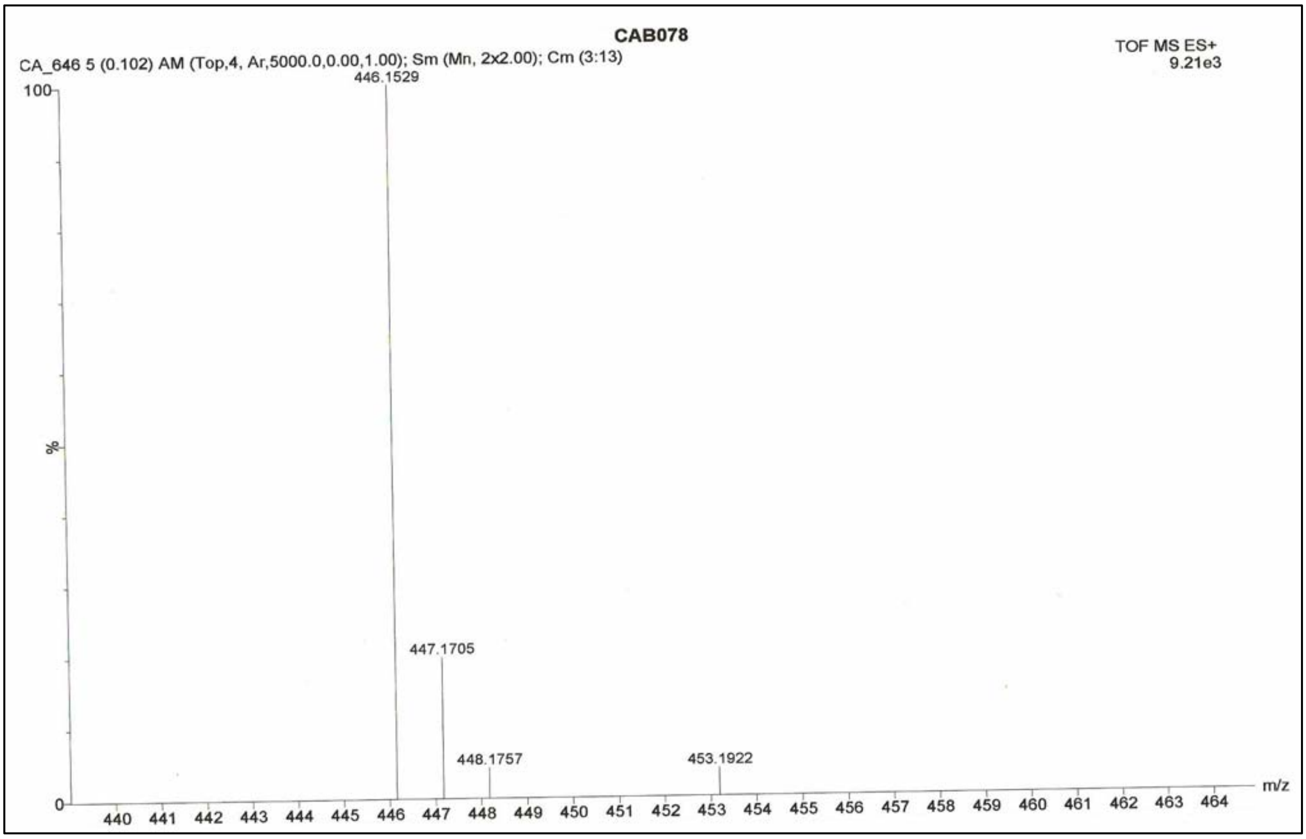

Supplement: Supplementary file 1 [file molecules-17-06100-s001.pdf]
